# Superelastic High‐Entropy Oxide Ceramic Aerogels for Thermal Superinsulation and Sealing at Extreme Conditions

**DOI:** 10.1002/advs.202516840

**Published:** 2025-12-03

**Authors:** Xiaoke Jiang, Tao Du, Hengzhong Fan, Junhong Liu, Yunfeng Su, Peng He, Hongxiang Chen, Litian Hu, Yongsheng Zhang, Qiangqiang Zhang

**Affiliations:** ^1^ State Key Laboratory of Solid Lubrication Lanzhou Institute of Chemical Physics Chinese Academy of Sciences Lanzhou 730000 P. R. China; ^2^ Center of Materials Science and Optoelectronics Engineering University of Chinese Academy of Sciences Beijing 100049 P. R. China; ^3^ Key Lab of Smart Prevention and Mitigation of Civil Engineering Disasters of the Ministry of Industry and Information Technology Harbin Institute of Technology Harbin 150090 P. R. China; ^4^ College of Civil Engineering and Mechanics Lanzhou University Lanzhou 730000 P. R. China; ^5^ Key Laboratory of Mechanics on Disaster and Environment in Western China (Lanzhou University) The Ministry of Education of China Lanzhou 730000 P. R. China; ^6^ School of Materials Science and Engineering Fujian University of Technology Fuzhou 350118 P. R. China

**Keywords:** high‐entropy ceramic, molecular synthesis route, nanofiber aerogels, superelasticity, synergistic interplay, thermal superinsulation

## Abstract

The lightweight ceramic aerogels are plagued by thermal instability and mechanical degeneration at extreme conditions. In this study, a high‐entropy oxide ceramic of (Gd_1/2_Lu_1/2_)_2_(Ti_1/3_Zr_1/3_Hf_1/3_)_2_O_7_ (GLTZH) is prepared through a molecular synthesis route of pyrolytic solid‐solution reactions. The atomic resolution observations visualize the phase transition of polyacetylacetonato metal complexes into a defect‐fluorite structured high‐entropy oxide after thermal treatment at 200 to 1100 °C. The GLTZH oxide demonstrates exceptional crystallographic stability without severe grain growth, and element segregation appeared under prolonged exposure to extremely high temperature (≈1500 °C). This originates from the intricate coupling mechanism among entropy‐driven lattice distortion, high‐entropy stabilization, and orbital hybridization effects. Furthermore, GLTZH‐based lightweight nanofiber aerogel is constructed through electrospinning and followed by thermal annealing at 1000 °C. This architectured high‐entropy ceramic aerogel manifests unprecedented thermomechanical properties, including superelastic compressibility of 98% from −196 to 1500 °C, and thermal superinsulation capacity (24.14 mW·m^−1^K^−1^ at room temperature, 81.21 mW·m^−1^K^−1^ at 1000 °C). Due to superior performances beyond most conventional ceramic counterparts, the high‐entropy GLTZH paves a new pathway for advanced ceramic aerogel design in thermal insulation across a wide temperature range, such as thermal protection of hypersonic aircraft.

## Graphical abstract

A high‐entropy oxide ceramic of (Gd_1/2_Lu_1/2_)_2_(Ti_1/3_Zr_1/3_Hf_1/3_)_2_O_7_ is prepared through a molecular synthesis route of pyrolytic solid‐solution reactions. The architectured nanofiber aerogel manifests unprecedented thermomechanical properties, including superelastic compressibility of 98% from −196 to 1500 °C, and thermal superinsulation capacity (24.14 mW·m^−1^ K^−1^ at room temperature, 81.21 mW·m^−1^ K^−1^ at 1000 °C). It suggests promising potential for thermal insulation across a wide temperature range.

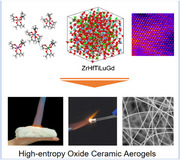

## 1 Introduction

Owing to their highly porous microstructure, low thermal conductivity, and corrosion resistance,^[^ ^1^, ^2^, ^3^ ^]^ lightweight ceramic aerogels are competitive candidates for thermal insulation under severe temperature variations and high‐temperature exposure in the military, aviation, and astronautic fields.^[^ ^4^, ^5^, ^6^, ^7^ ^]^ Conventional ceramic aerogels are composed of either oxides or non‐oxide compounds, such as Al_2_O_3_, ZrO_2,_ T_i_O_2,_ SiO_2_, BN, and SiC.^[^ ^8^, ^9^, ^10^, ^11^, ^12^, ^13^, ^14^ ^]^ However, the inherent plagues between mechanical brittleness (e.g., covalent bond) and thermal instability (e.g., severe grain growth) of single‐principal‐element metal oxide are prone to cause the microstructural failure under intense thermal shock or large deformation. These compulsive transitions to crystalline states or lattice phases severely constrain applications of ceramic aerogels for thermal protection under extreme thermal environments.^[^ ^4^ ^]^ It is still a great challenge to create ceramic aerogels with a prominent combination of highly mechanical robustness, predominant insulation capacity, and thermal stability.^[^ ^1^, ^12^ ^,^ ^15^ ^]^

High‐entropy materials have gained extensive attention because of their superior mechanical and thermal performance.^[^ ^16^, ^17^ ^]^ For instance, diffusion retardation effectively suppresses severe grain growth at high temperatures,^[^ ^18^, ^19^, ^20^ ^]^ while lattice distortion significantly enhances the phonon scattering and thermal insulation capacity.^[^ ^21^, ^22^ ^]^ High‐entropy effect offers a credible inspiration for ceramic aerogels overcoming the conflict between mechanical and thermal performances. Among them, A_2_B_2_O_7_ oxides, as one of the most representative candidates have been widely used as a thermal barrier in gas turbine engines due to their excellent thermal stability.^[^ ^23^ ^]^ In general, rare‐earth (+3 valence) or alkaline earth metal cations (+2 valence) are selected as A‐site cations, while either +4 or +5 valence metal cations are located at the B‐site.

Among diverse synthesis methods,^[^ ^24^, ^25^, ^26^ ^]^ electrospinning has been widely employed to create fiber‐based ceramic aerogels beyond the geometric limitation of chemical reacting mold.^[^ ^27^ ^]^ Metal salt precursors require high copolymer content for spinnability, yet subsequent polymer removal during heat treatment introduces defects that deteriorate mechanical performance. Comparatively, a linearly structured precursor exhibits better spinnability and improved mechanical flexibility.^[^ ^4^, ^28^ ^]^ Besides, a 3D porous carbon framework is selected as the template to obtain ceramic‐composed aerogels (e.g., MXene) for electromagnetic wave absorbing and pressure sensing applications.^[^ ^29^, ^30^, ^31^ ^]^ Usually, the sol–gel method combined with supercritical drying is conducted to prepare high‐entropy ceramic (HEC) aerogels, such as (YErYbGdLa)_2_Zr_2_O_7_^[^ ^32^ ^]^ and (YErYbLu)_2_SiO_5_.^[^ ^33^ ^]^ Even though sol–gel synthesized HEC aerogels have demonstrated competitive performance on thermal insulation with thermal conductivity down to 0.0263 W·m^−1^·K^−1^, they are still faced with high brittleness and poor compressive resilience because of their pearl‐necklace‐like microstructure.^[^ ^34^, ^35^ ^]^

In this study, we synthesized a defect‐fluorite structured high‐entropy oxide of (Gd_1/2_Lu_1/2_)_2_(Ti_1/3_Zr_1/3_Hf_1/3_)_2_O_7_ (simplified as GLTZH). Atomic‐resolution characterization confirms the transition from noncrystalline (≈400 °C) and hypocrystalline (≈600 °C) to single‐phase crystal (≈800 °C). GLTZH retains ultrafine grain for either 1000 °C treatment (≈10 nm) or 1400 °C exposure (≈240 nm). The crystallographic stability arises from a synergistic triad of configuration entropy regulation, such as severe lattice distortion and cationic diffusion suppression. Leveraging this atomic‐scale design framework, ultralight high‐entropy oxide nanofiber aerogels are successfully prepared via an electrospinning process. The resulting GLTZH aerogel exhibits highly porous hierarchical architecture and unparalleled thermomechanical resilience, including superelastic compressibility (≈98%) from −196 to 1500 °C, and ultralow thermal conductivity (24.14 mW·m^−1^·K^−1^ at 25 °C; 81.21 mW·m^−1^·K^−1^ at 1000 °C). This study opens a pathway for rational design of protective ceramics with outstanding thermal stability and mechanical resilience under extreme environments.

## 2 Result and Discussion

### 2.1 Structural Design of GLTZH

**Figure 1:**
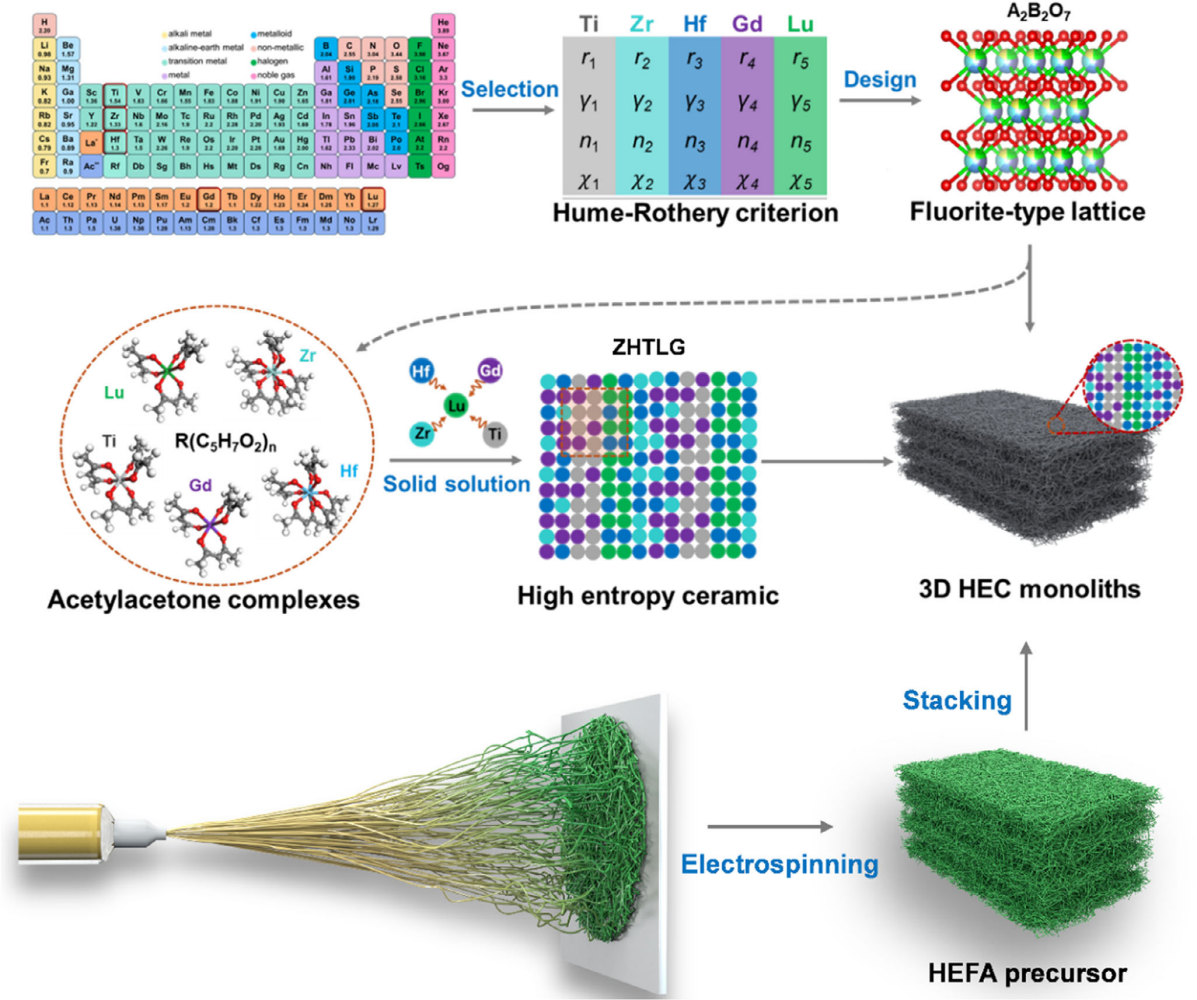
The schematic illustration of conceptual formation for high‐entropy oxide‐based ceramic aerogels, including metal cation selection, proportion optimization, the crystal design, the acetylacetone complex synthesis, the solid‐solution reaction, and 3D aerogel monolith stacking.

As shown in **Figure** **1**, the molecular synthesis routine aims to prepare high‐entropy oxide of A_2_B_2_O_7_ and related aerogel monoliths. Specifically, considering the influences of ionic radius,^[^ ^36^, ^37^ ^]^ crystal structure,^[^ ^38^ ^]^ electron concentration,^[^ ^39^ ^]^ electronegativity^[^ ^18^, ^39^ ^]^ on solid solution formation, five high‐melting‐point metallic elements (Gd, Lu, Ti, Zr, Hf) are primarily selected from the huge element space according to the Hume‐Rothery criterion^[^ ^40^ ^]^ after compared with the other compositions of ZrHfTiGdLa, ZrHfTiLaY, and ZrHfTiCeSm (Figure S1 and Tables S1 and S2, Supporting Information). ZrHfTiLuGd exhibits the lowest potential energy over time and the greatest thermodynamic stability compared to the other three phases. The combination of trivalent (Gd^3+^, Lu^3+^) and tetravalent (Hf^4+^, Ti^4+^, Zr^4+^) cations maintains overall charge neutrality (O^2−^ anions) while maximizing cationic configurational entropy, which enables to stabilization of a single solid‐solution phase at high temperature. The ionic radii of these cations span a moderate range (e.g., Gd^3+^ ≈ 1.053 Å; Lu^3+^ ≈ 0.977 Å; Ti^4+^ ≈ 0.745 Å; Zr^4+^ ≈ 0.84 Å; Hf^4+^ ≈ 0.83 Å). This promotes local lattice distortion that enhances phonon scattering, leading to greater thermal stability while still maintaining a single‐phase solid‐solution and low thermal conductivity. The more detailed design process is provided in the Supporting Information. After that, five polyacetylacetonato metal complexes ([R(C_5_H_7_O_2_)_x_]_n_) were synthesized through the chemical reaction of Equation (1). Hereinto, Gd and Lu, as rare earth elements, are positioned at the A‐site, while Zr, Hf, and Ti, as transition metals, are chosen for the B‐site. The obtained high‐entropy oxide of (Gd_1/2_Lu_1/2_)_2_(Ti_1/3_Zr_1/3_Hf_1/3_)_2_O_7_ was simplified to note as GLTZH.

XRD refinement spectra in Figure S2a (Supporting Information) indicate that the GLTZH annealed at 1000 °C exhibits characteristic superlattice diffraction peaks of a typical single‐phase defective fluorite structure.^[^ ^28^ ^]^ The specific refined parameters are shown in Tables S3 and S4 (Supporting Information). As shown in Figure S2b (Supporting Information), four characteristic peaks (E_g_, A_1g_, and F_2g_(2)) corresponded to the pyrochlore structure, demonstrating that the transition of A‐site/B‐site cations and oxygen vacancies from disorder to order distribution of the GLTZH as long as the calcination temperature is elevated over 1000 °C.^[^ ^41^ ^]^ The multi‐component polyacetylacetonato metal complexes are transferred to the high‐entropy oxide GLTZH via a solid‐solution reaction after being uniformly mixed in a stoichiometric ratio. Subsequently, GLTZH nanofibers were assembled into a highly porous HEC aerogel via electrospinning for thermal insulation applications in extreme environments.

(1) $$\begin{array}{c} nZrOCl_{2}\cdot 8H_{2}O+C_{5}H_{8}O_{2}+C_{6}H_{15}N\to {\left[Zr{\left(C_{5}H_{7}O_{2}\right)}_{2}\right]}_{n}\\ \;+C_{6}H_{15}NHCl+H_{2}O\\ nHfOCl_{2}\cdot 8H_{2}O+C_{5}H_{8}O_{2}+C_{6}H_{15}N\to {\left[Hf{\left(C_{5}H_{7}O_{2}\right)}_{2}\right]}_{n}\\ \;+C_{6}H_{15}NHCl+H_{2}O\\ nTi{\left(OC_{4}H_{9}\right)}_{4}+C_{5}H_{8}O_{2}\to {\left[Ti{\left(C_{5}H_{7}O_{2}\right)}_{4}\right]}_{n}+C_{4}H_{9}OH\\ nGdCl_{3}\cdot 6H_{2}O+C_{5}H_{8}O_{2}+C_{6}H_{15}N\to {\left[Gd{\left(C_{5}H_{7}O_{2}\right)}_{3}\right]}_{n}\\ \;+C_{6}H_{15}NHCl+H_{2}O\\ nLuCl_{3}\cdot 6H_{2}O+C_{5}H_{8}O_{2}+C_{6}H_{15}N\to {\left[Lu{\left(C_{5}H_{7}O_{2}\right)}_{3}\right]}_{n}\\ \;+C_{6}H_{15}NHCl+H_{2}O \end{array}$$

### 2.2 Phase Transformation Mechanism of GLTZH

**Figure 2:**
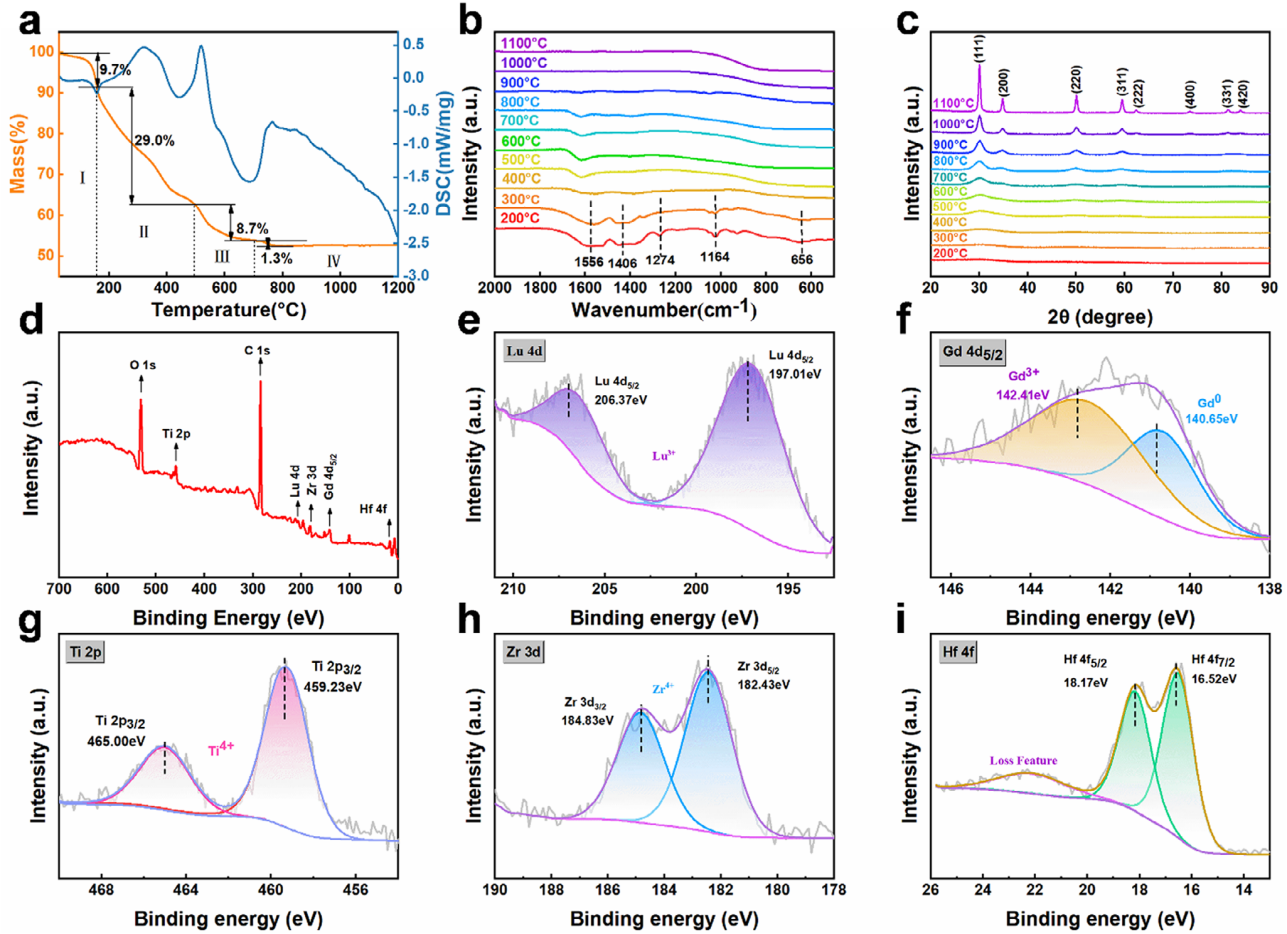
The comparative surveys of chemical components and crystal structure evolution. a) thermogravimetric analysis‐differential scanning calorimetry of acetylacetone complex precursor. b) Fourier‐transform infrared spectroscopy and c) X‐ray diffraction of the multi‐component precursor treated at different temperatures from 200 to 1100 °C. d) Full‐spectrum binding energy of XPS for GLTZH prepared at 1000 °C. e–i) The related deconvolution spectra survey for Zr 3d, Hf 4f, Gd 4d, Ti 2p, and Lu 4d, respectively.

As shown in **Figure** **2**, the dynamic behavior and chemical composition evolution of the acetylacetone complex pyrolysis are jointly characterized to understand phase transition through thermogravimetric analysis‐differential scanning calorimetry (TG‐DSC), Fourier‐transform infrared spectroscopy (FT‐IR), X‐ray diffraction (XRD), electron paramagnetic resonance (EPR), and X‐ray photoelectron spectroscopy (XPS). Specifically, TG‐DSC perceives the notable mass loss of approximately 48.7% with a crystalline change appearing at 750 °C. It features the transformation sign from a noncrystalline phase to a crystalline structure (**Figure** **2**a). The related color changes of the multi‐component precursor from yellow, black, and white mark the pyrolysis process under different temperatures (see Figure S3, Supporting Information). FT‐IR spectroscopy in Figure 2b exhibits distinct absorption peaks of acetylacetone‐metal complexes at 1556, 1406, 1274, and 1164 cm^−1^,^[^ ^28^ ^]^ while the diminished peaks for C─H, C─C, and CH_3_ bonds demonstrate the complete decomposition of acetylacetone complexes beyond 600 °C. Simultaneously, a new absorption peak at 656 cm^−1^ reflects the formation of metal‐oxygen bonds and the gradual propagation of the oxide network.^[^ ^42^ ^]^

Figure 2c displays the dynamic behavior of phase structural transition during precursor pyrolysis from 200 to 1100 °C. The emergence of a new diffraction peak at 30° for the crystal face (111) on XRD patterns implies that the fluorite‐type structure of the GLTZH starts to form after the temperature exceeded 600 °C. Additionally, the O1s spectra of GLTZH are deconvoluted into four fitting peaks (529.61, 531.05, 532.27, and 533.09 eV) (see Figure S4a, Supporting Information). The peak centered at 531.05 eV corresponds to oxygen‐deficient regions such as oxygen vacancies (O_V_).^[^ ^43^ ^]^ Similarly, the EPR spectrum exhibits a distinct peak at *g* = 2.003, which suggests a similar presence of oxygen vacancies as XPS detection (see Figure S4b, Supporting Information). Moreover, the diffraction peaks tend to be sharper as the temperature increases from 600 to 1100 °C, quantifying the crystallization of acetylacetone complexes converting into a single‐phase high‐entropy oxide from a hypocrystalline state.

As shown in Figure S5 (Supporting Information), selected‐area electron diffraction patterns for GLTZH confirm the stability of the single‐phase disordered defective fluorite structure after 1100 °C annealing treatment. Moreover, the full‐spectrum binding energy of XPS signals for GLTZH prepared at 1000 °C in Figure 2d unambiguously identifies five characteristic peaks at 459.00, 17.20, 183.08, 207.00, and 141.00 eV corresponding to Zr 3d, Hf 4f, Ti 2p, Lu 4d, and Gd 4d_5/2_ orbitals. Figure 2e–i illustrates the deconvolution spectra for each metallic element, which reveals exclusively singular binding energy components associated with the respective M─O bond. These spectrochemical observations confirm the absence of intermetallic solid‐solution reactions during phase conversion from amorphous to crystallization states.

### 2.3 Dynamic Behavior of GLTZH Microstructural Formation

**Figure 3:**
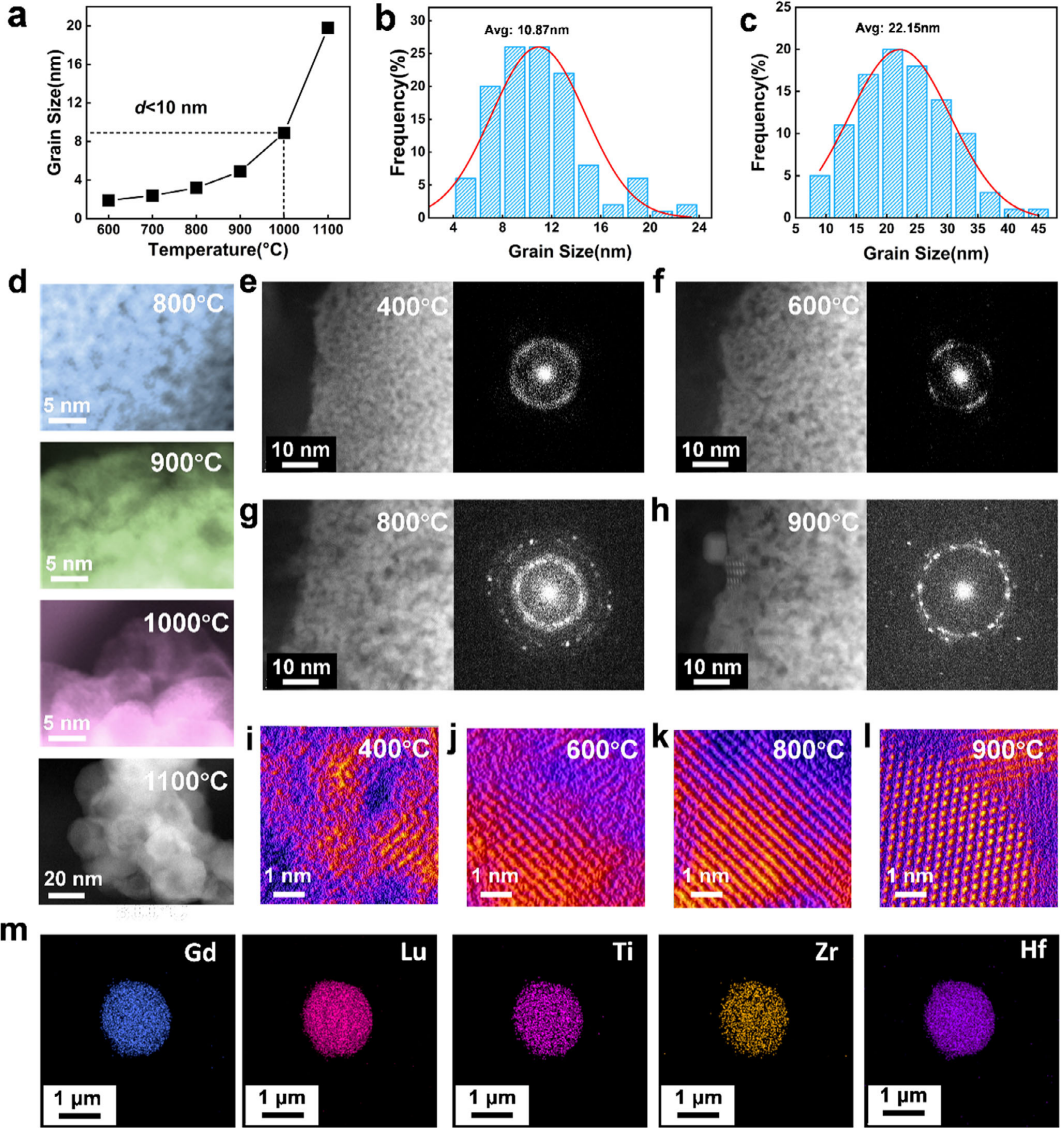
The dynamic mechanism of GLTZH formation. a) Average grain size calculated from XRD. Grain size distribution after annealing at b) 1000 °C, and c) 1100 °C. d) The evolution of the morphology imaged in STEM. Atomic‐resolution HAADF‐STEM and FFT images after annealing at e) 400 °C, f) 600 °C, g) 800 °C, and h) 900 °C. Atomic‐scale evidence of polymer‐to‐ceramic phase conversion at different temperatures of i) 400 °C, j) 600 °C, k) 800 °C, l) 900 °C, and m) 1000 °C. n) Element distribution mapping for Zr, Hf, Ti, Lu, and Gd.

To deeply understand the transition behavior of GLTZH single‐phase fluorite crystallization, high‐angle annular dark‐field scanning transmission electron microscopy (HAADF‐STEM) is selected to trace the phase evolution of the precursor transformation. As shown in **Figure** **3**a, the average grain size is calculated from the XRD patterns based on the Scherrer equation.^[^ ^44^ ^]^ As the temperature elevates from 600 to 1100 °C, the grain size enlarges from 2.4 to 19.8 nm. This is consistent with the TEM observations that reveal grain sizes retaining around 10 and 20 nm at 1000 and 1100 °C, respectively (Figure 3b,c; Figure S6, Supporting Information). The STEM images in Figure 3d further clarify the evolution feature of the morphologies under different temperatures (Figure S7, Supporting Information). Specifically, a faint diffraction ring in the fast Fourier transform (FFT) pattern combined with a diffuse diffraction peak for the (111) crystal plane in XRD signifies the initiation of nano‐sized crystalline grain formation at 400 °C (Figure 3e). The color transition from yellow to black at this stage implies significant carbonization and decomposition (Figure S3, Supporting Information). As the temperature elevated up to 600 °C, micropores prompt oxygen diffusion and further growth of crystalline grains (Figure 3f). Meanwhile, the transition around 750 °C is primarily associated with the structural ordering and coordination environment adjustment of the inorganic components rather than any changes in oxidation state, which is jointly validated by the comprehensive characterization of TG‐DSC, XRD, and XPS (Figure 2a,c; Figure S8, Supporting Information) at 700 and 800 °C, respectively. The discrete diffraction spots in FFT images for temperature over 800 °C show progressive improvement of order degree and crystallization (Figure 3g‐h).^[^ ^45^ ^]^

Atomic resolution observations in Figure 3i–l further validate the lattice structural evolution of the acetylacetone complexed precursor. It is in an amorphous state without significant volume changes below 200 °C (Figure S9, Supporting Information), while local nucleation and nanocrystal formation occur as the temperature increases up to 400 °C (Figure 3i). The transition from the disordered state to the oriented high‐entropy ceramic phase is achieved for temperatures ranging from 600 to 800 °C (Figure 3j,k). Micropores generated during polymer decomposition are progressively eliminated during grain growth. As long as the temperature is beyond 900 °C, a highly crystalline GLTZH forms with a single‐phase defective fluorite structure (Figure 3l,m). The emergence of distinct peaks at 5.3 and 6.2 nm^−1^ in the FFT‐derived power spectra provides additional evidence for the formation of a disordered fluorite phase (Figure S10, Supporting Information).^[^ ^46^, ^47^ ^]^ The phase evolution of GLTZH proceeds through three distinct stages: i) an amorphous state below 200 °C, ii) ordered transformation between 400–600 °C, and iii) high crystallization above 800 °C (Figure S11, Supporting Information).

Moreover, the energy‐dispersive X‐ray spectroscopy (EDS) maps in Figure 3n and Figure S12 (Supporting Information) cooperatively demonstrate the uniform distribution features of Gd, Lu, Ti, Zr, and Hf. Multi‐components eventually form the thermally stable phase of high‐entropy ceramic without segregation or aggregation after overcoming thermodynamic disturbances under temperatures ranging from 400 to 1100 °C. The combined reduction of atomic diffusion distance and nucleation barrier promotes the formation of GLTZH nanoclusters or nuclei, facilitating atomic‐level mixing of the multiple elements (Gd, Lu, Ti, Zr, and Hf).^[^ ^48^, ^49^ ^]^ Compared with conventional high‐temperature solid‐state sintering, the acetylacetone complex precursor‐derived molecular synthesis pathway eliminates compositional segregation and grain boundary impurities that are typically induced by high‐energy ball milling.^[^ ^50^ ^]^

### 2.4 Thermal Stability of GLTZH High‐Entropy Oxide

**Figure 4:**
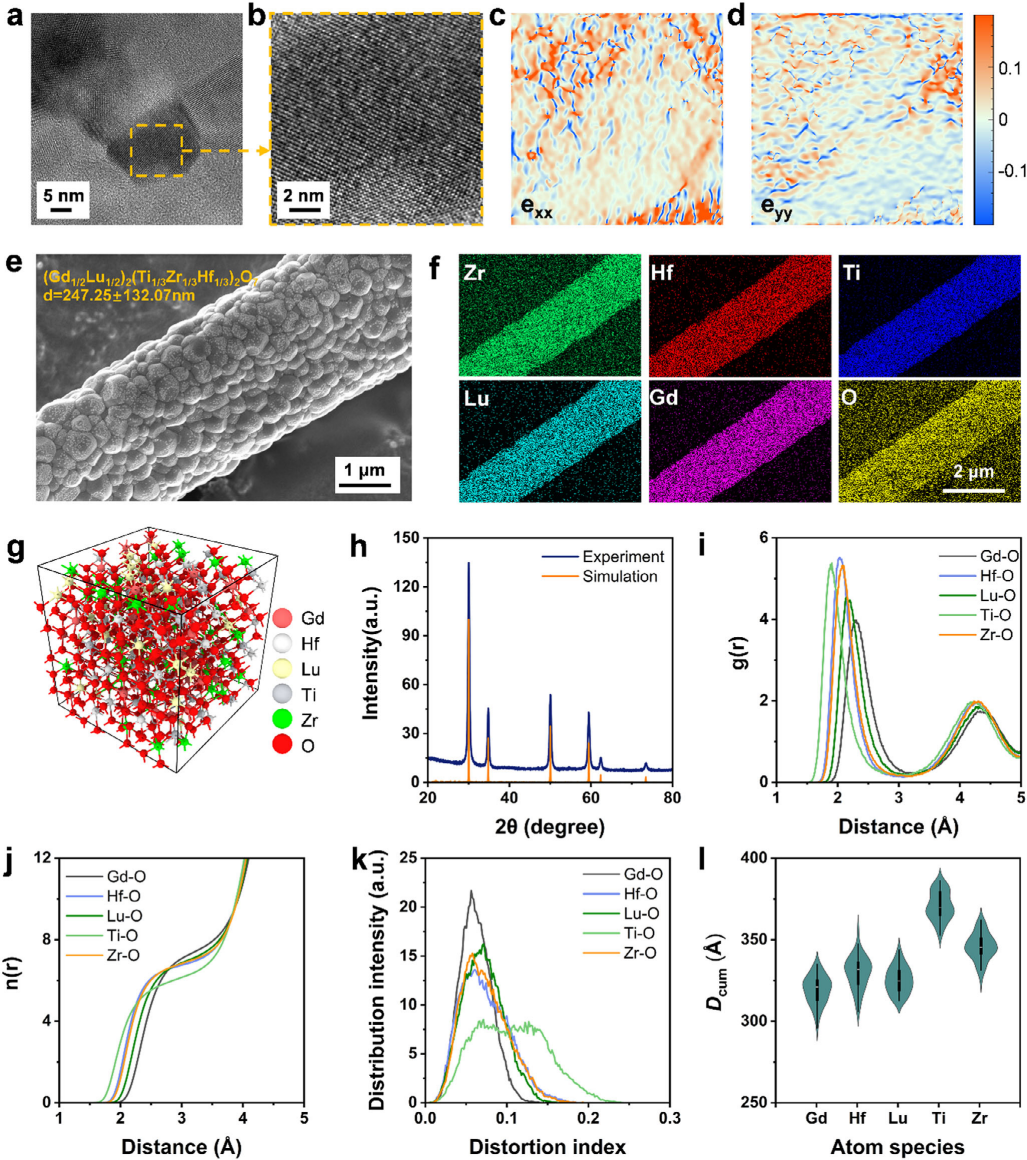
a,b) HRTEM images of GLTZH. c,d) GPA mapping along the xx and yy directions, respectively. e) Grain sizes of GLTZH annealed at 1400 °C for 2 h. f) EDS images of GLTZH annealed at 1400 °C. g) Atomic snapshot of the initial structure for AIMD simulation. h) Comparison of the simulated XRD with the experiment. i) Partial pair distribution functions of the atom pairs. j) Running coordination number of the cations of the HEC structure. k) Distortion index of the polyhedra in the GLTZH structure under 2073 K simulation. l) Cumulative non‐affine displacement of the cations after AIMD simulation.

To further investigate the influence of multi‐principal cations on the thermal stability of GLTZH oxide, local lattice strain is further analyzed using geometric phase analysis (GPA).^[^ ^51^ ^]^ High‐resolution TEM images in **Figures** **4**a,b and S13 (Supporting Information) demonstrate the obvious lattice distortion as typical high‐entropy effects that originate from the disordered atomic occupation on A/B sites of five cations (Gd, Lu, Ti, Zr, Hf). It results in widespread distribution of lattice strain along the ε_xx_ and ε_yy_ directions (see Figure 4c,d).^[^ ^52^ ^]^ The blue regions correspond to compressive strain (‐), while the red regions indicate the tensile strain (+). The tensile strain is primarily attributed to the smaller ionic radius of Ti (0.745 Å), which becomes elongated within the lattice as long as it accommodates larger neighboring atoms. Compressive strain is predominantly induced by Lu and Gd, which possess larger ionic radii (1.053 Å and 0.977 Å, respectively), leading to localized lattice expansion. In contrast, Zr (0.84 Å) and Hf (0.83 Å) with intermediate ionic radii exhibit minimal amplitude of either compressive or tensile strain. Table S4 (Supporting Information) displays the crystal structures and unit cell parameters of the individual oxide components. Significant changes in the unit cell parameters are observed when these components are dissolved into the same lattice. This underscores the pivotal role of the atomic size mismatch of HEC in generating complex lattice distortions. To quantify the changes in bond lengths and angles induced by lattice distortion, 4×4×4 supercell models of ZrO_2_, HfO_2_, and GLTZH were constructed as shown in Figure S14 (Supporting Information). The numerical simulation clearly indicates that TM‐O bond lengths (e.g., Hf‐O: 2.172 Å→ 2.121 Å) and ∠TM‐O‐TM angles (e.g., ∠O‐Hf‐O: 70.52°→ 77.71°) have undergone significant lattice distortion in GLTZH, which is accompanied by changes in unit cell volume and shape.

Within high‐entropy GLTZH ceramics, the atom deviations from ideal positions induce high‐density lattice distortion that improves mechanical properties, impedes grain growth, and sluggish atom diffusion.^[^ ^53^ ^]^ For the sake of appraisal, the grain growth behaviors of single‐component, binary, and HEC fibers are comparatively surveyed after thermal exposure at 1400 °C for 2 h. As shown in Figure 4e, the average grain size of the GLTZH remains within 250 nm, whereas ZrO_2_ and yttria‐stabilized zirconia (YSZ) have much larger grain sizes of 625 and 448 nm, respectively (Figure S15, Supporting Information). High‐entropy GLTZH ceramics exhibit a more stable defective fluorite structure without elemental segregation under high‐temperature conditions, rather than those of conventional ceramics (Figure 4f). This sluggish diffusion effect is attributed to the low variation of Gibbs free energy (Δ*G*_mix_) and the high migration energy barrier at grain boundaries resulting from high‐density lattice distortions.

To quantitatively investigate the atomistic origin of the thermal stability of ZHTLG ceramic, ab initio molecular dynamics simulations (AIMD) under 2073K are subsequently performed, as shown in Figure 4g–l. The established lattice model of GLTZH maintains the same XRD pattern as the experimental data to confirm the structural accuracy (Figure 4h). The partial pair distribution functions of the cation–oxygen pairs in Figure 4i highlight variations in bond lengths arising from differences in cation sizes. The running coordination number in Figure 4j indicates that most cations are approximately seven‐coordinated within the first coordination shell (*r* = 3.1 Å) except for Ti. The relatively lower slope of the Ti─O coordination curve with distance also suggests the presence of multiple coordination states, attributed to the degeneration of Ti─O bonds over 1800 °C in theory. As shown in Figure 4k, the Ti─O polyhedra exhibit greater distortion than those of other cations, indicating that the Ti─O polyhedra primarily collapse in response to the onset of melting in GLTZH at 1800 °C.

Table S6 (Supporting Information) indicates that the melting point of TiO_2_ (≈1800 °C) is significantly lower than that of the oxides of the other four elements (>2300 °C), which reflects the bottom line of the serving temperature of GLTZH. To further probe the propensity of the different cations’ arrangements, the metric of cumulative non‐affine displacement (*D*_cum_) was selected to quantitatively evaluate the deviation from the average, non‐rigid motion of the system (Figure 4l). Compared to the conventional mean‐squared displacement, *D*_cum_ is more commonly used to describe the diffusion of a collection of atoms and capture the dynamic behavior of individual atoms under thermal fluctuations (Figure S16, Supporting Information). *D*_cum_ confirms that Ti has the most pronounced dynamic behavior among all cations, even though they are primarily introduced to suppress infrared radiation at high temperatures. To further understand the structural stability mechanism in a broader thermal range, the AIMD simulation was extensively conducted at a lower temperature of 1273 K (Figure S17, Supporting Information). The parameters featuring structure evolution demonstrate a highly coincident tendency with 2073 K, indicating a higher propensity for Ti atoms to arrange under thermal activation.

### 2.5 Functionalizing GLTZH High‐Entropy Ceramic Aerogel

**Figure 5:**
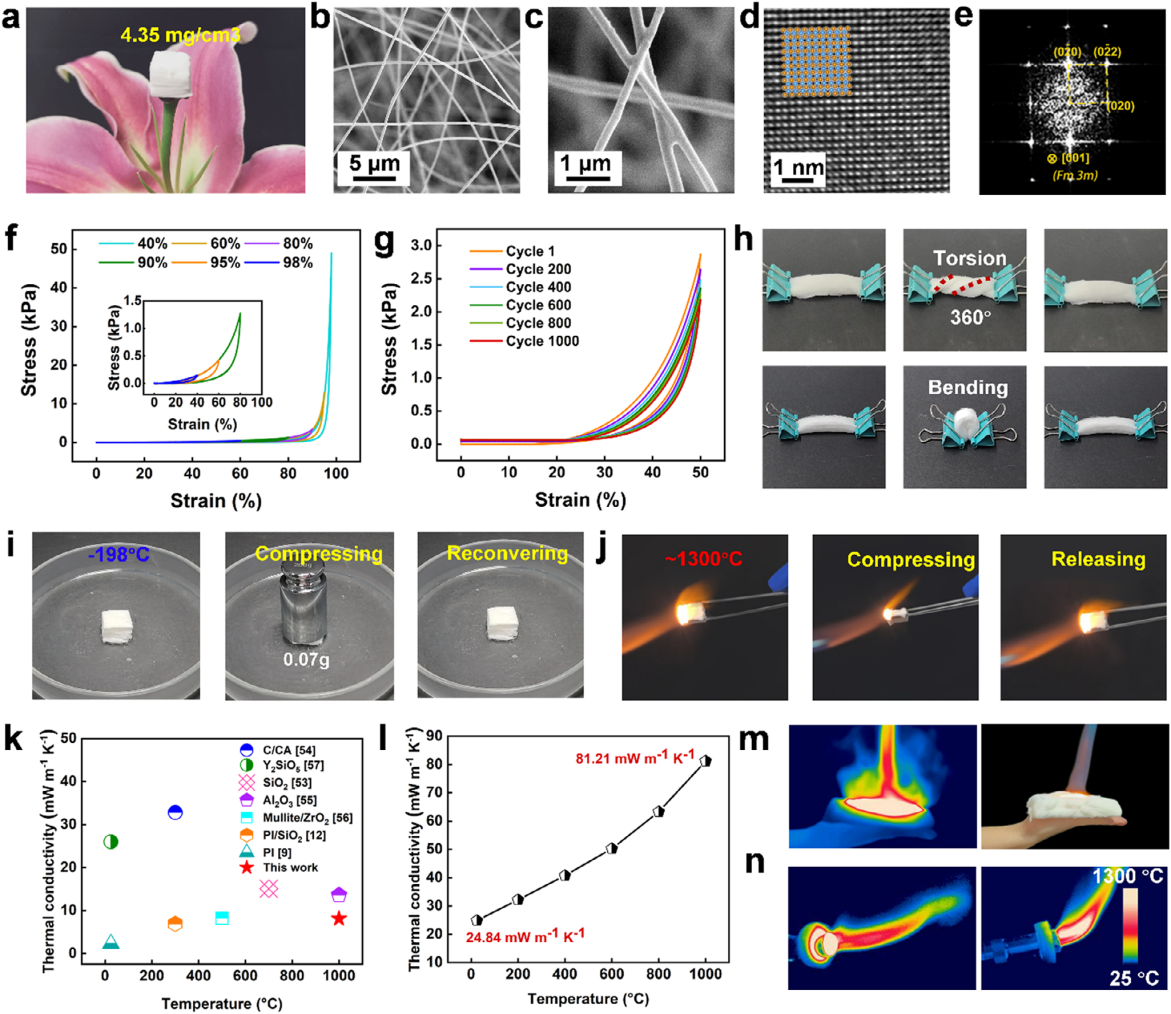
The mechanical and thermal properties of GLTZH high entropy ceramic fiber aerogel. a) The lightweight sample freely standing over the flower petals. b,c) The porous morphology at different magnifications. d) Atomic resolution lattice image. e) FFT image of (d). f) The uniaxial compression with maximum engineering strain up to 98%. g) A 1000 cyclic compression under engineering strain of 50%. h) The remarkable deformability under torsion and bending schemes, respectively. i) The ultralarge compaction in liquid nitrogen at −198 °C. j) The superelastic deformation under extremely high temperature of ≈1300 °C. k) Comparative thermal conductivity at different temperatures. l) Comparison of thermal conductivity for different insulating materials. m,n) The thermal insulation and sealing applications to the human body and simulated engine nozzle under extremely hot conditions.

To leverage the unique advantages of high‐entropy ceramics in mechanical and thermal properties, GLTZH aerogel‐like monoliths are fabricated via an electrospinning process of five polyacetylacetonato metal complexes (Gd, Lu, Ti, Zr, and Hf) (see Figure 1). The aerogel density was controlled to ensure structural uniformity and batch consistency for scalable fabrication of aerogel samples with diverse sizes (Figure S18, Supporting Information). The resulting highly porous aerogel exhibits a lightweight density of 4.35 mg/cm^3^, enabling it to freely stand over the flower petals (**Figure** **5**a). As shown in Figure 5b‐c, GLTZH nanofibers (average diameter: 250 nm) form a hierarchical network with an interlayer spacing of ≈5 µm (Figure S19a, Supporting Information). The nodes are strongly bonded to enhance structural robustness and fatigue resistance under thermal stimuli or large deformations. The grain characteristics of a typical region in high‐entropy oxides reveal that the lattice fringes of 0.254 and 0.294 nm corresponded to the (111) and (200) facets (Figure S19b,c, Supporting Information). Figure 5d,e exhibits an FFT image with the space group of a single‐phase high‐entropy solid solution belonging to *Fm3m*. In terms of lattice structure, it presents a cubic symmetry with the (020) and (−200) crystallographic planes visible along the [001] zone axis. The SAED patterns validate that four diffraction rings corresponding to the (111), (200), (220), and (311) planes, confirming the defective fluorite structure in GLTZH nanofiber (Figure S19d, Supporting Information). Moreover, EDS mappings of nanofibers demonstrate a uniform distribution of Zr, Hf, Ti, Lu, and Gd without any elemental segregation (Figure S19e, Supporting Information).

The mechanical and thermal insulating properties of GLTZH high entropy oxide aerogels are investigated under various deformation schemes and service conditions. The constant displacement rate is 10 mm min^−1^, which corresponds to a strain rate of 0.011 s^−1^. As shown in Figure 5f, it displays the superelastic deformability with a recoverable compressive engineering strain as large as 98% (Movie S1, Supporting Information). Moreover, after heat treatment at 1500 °C for 5–30 min, the GLTZH aerogel retains its structural integrity without any fracture appearing, which even exhibits near‐complete recovery from 90% compression (Figure S20, Supporting Information). This is attributed to the highly flexible and resilient nanofibers and strong interfaces. The cooperative effect between the inter‐fiber bonding strength and the good flexibility of individual fibers provides the internal driving force at the microscale to permit the large compression deformation. The structural stability and fatigue resistance are further verified under various deformation schemes, including 1000 cyclic compression (50% engineering strain), 360° torsion, and 180° fold (Figure 5g,h; Movies S2 and S3, Supporting Information). Moreover, the GLTZH aerogel maintains its remarkable compressibility even under extremely cryogenic conditions. The highly compacted sample could fully recover its original configuration after being immersed in liquid nitrogen (−198 °C) (Figure 5i; Movie S4, Supporting Information). Concomitantly, it exhibits ultrahigh flexibility when it is exposed to an extremely high temperature of an acetylene torch. Under the dynamic flaming treatment over 1300 °C, the GLTZH aerogel could be compacted into a thin pancake‐like shape and completely recovered to its original shape without structural fracture (Movie S5, Supporting Information). It reveals the high stability of resilient aerogels’ structure from individual nanofibers to a porous network.

Moreover, the highly porous microstructure, high configurational entropy, and severe lattice distortion make the ceramic fiber aerogel promising for thermal insulation applications. As illustrated in Figure 5k, GLTZH aerogel presents a comparatively higher serving temperature (≈1300 °C) as well as much lower thermal conductivity (24.84 mW·m^−1^K^−1^ at room temperature, 81.21 mW·m^−1^K^−1^ at 1000 °C) than that of either organic fiber aerogels (e.g., PI, PI/ SiO_2_) or other ceramic aerogels (e.g., SiO_2_, Carbon, Al_2_O_3_, Mullite/ZrO_2_, Y_2_SiO_5_).^[^ ^9^, ^12^, ^54^, ^55^, ^56^, ^57^, ^58^ ^]^ Direct comparison in Figure S21 and Table S7 (Supporting Information) further validates the priority of GLTZH on thermal conductivity at 1000 °C. As presented in Figure 5l, the thermal conductivity shows an approximately linear dependence (*y* = 0.056*x* + 20.501) on temperature from room temperature to 1000 °C with the corresponding linear fitting results presented in Figure S22 (Supporting Information). Besides the enhanced phonon scattering and constrained air heat transfer, the thermal superinsulation at high temperature mostly originates from the solid solution of TiO_2_ due to effective inhibition of infrared radiation.^[^ ^16^, ^59^ ^]^ As a result, the GLTZH nanofiber aerogel makes the temperature on the other side endurable for human hand (<50 °C) with the right side heating up to 1300 °C (Figure 5m; Movie S6, Supporting Information). It even presents excellent performance on thermal sealing of a simulated engine nozzle with center temperature over 1300 °C (Figure 5n). This validated the promising potential of high entropy oxide aerogel for thermal protection under extreme environments for industrial equipment, spacecraft, human body and air vehicle.

## 3 Conclusion

This study highlights a high‐entropy oxide GLTZH ceramic that is fabricated through a solid solution reaction after pyrolytic decomposition of multiple polyacetylacetone metal complexes by an equimolar ratio. Atomic resolution TEM observation confirms phase transition from amorphous state to ordered defect‐fluorite structure after 800 °C. GLTZH aerogel presents crystallographic stability with grain sizes retained ultrafine level even under prolonged exposure at 1400 °C, which surpasses coarsening limits of conventional oxide ceramics. This exceptional performance originates from the severe lattice distortion and suppressed cation diffusion because of the high configuration entropy effect. The GLTZH nanofiber aerogel is further constructed by electrospinning process, which exhibits a sequence of superior performances including lightweight density (4.35 mg cm^−3^), 98% compressible elasticity, ultralow thermal conductivity (24.14 mW·m^−1^·K^−1^ at 25 °C; 81.21 mW·m^−1^·K^−1^ at 1000 °C) and remarkable flame shielding capacity (≈1300 °C). These properties mainly arise from defect‐fluorite lattices with high configuration entropy that inhibit phonon propagation yet compromise mechanical deformation. This work establishes a new pathway via configuration entropy regulation to resolve the long‐term trade‐off between thermal stability and mechanical compliance in ceramics, making it promising for extreme‐environment applications (≈1500 °C), such as thermal protection systems in hypersonic vehicles, aero‐engine, and next‐generation insulation under harshly coupled conditions of thermal, mechanical, and oxidative stresses.

## 4 Experimental Section

### Materials

The following reagents are purchased for synthesizing high‐entropy ceramic aerogels, including ZrOCl_2_·8H_2_O, HfOCl_2_·8H_2_O, C_16_H_36_O_4_Ti, GdCl_3_·6H_2_O, LuCl_3_·6H_2_O (Shanghai Macklin Biochemical Technology Co., Ltd.), anhydrous methanol (CH_3_OH) (AR, ≥99.7%, Sinopharm Chemical Reagent Co., Ltd.), ethyl alcohol (C_2_H_5_OH) (AR, ≥99.7%, Damao Chemical Reagent Factory Co., Ltd.), acetylacetone (C_5_H_8_O_2_), triethylamine (C_6_H_15_N) (AR, ≥99.7%, Shanghai Macklin Biochemical Technology Co., Ltd.), polyethylene oxide (HO(CH_2_CH_2_O)_n_H) (PEO, *M*_W_ = 1000 000, Shanghai Aladdin Biochemical Technology Co., Ltd.), ethylenediamine (C_2_H_8_N_2_) (AR, ≥99.7%, Yantai Shuangshuang Chemical Co., Ltd.).

### Synthesis of High‐Entropy Oxide GLTZH Aerogel

Primarily, rare‐earth chloride salts (RECl_3_, RE = Lu, Gd) or metal chloride oxides (ZrOCl_2_⋅8H_2_O, HfOCl_2_⋅8H_2_O) are dissolved in methanol. Acetylacetone and triethylamine are subsequently added in a molar ratio of 1:1:3 (metal source: acetylacetone: triethylamine). After 2 h stirring, the clear solution is dried under reduced pressure at 50 °C to collect a polymer precursor containing triethylamine hydrochloride. Eventually, polyacetylacetone complexes (Zr, Hf, Lu, and Gd) are yielded through rotary evaporation at 40 °C from a clear solution. The reaction proceeds under atmospheric pressure, with triethylamine added dropwise under an ice bath condition (≈0 °C). Polyacetylacetone titanium (Ti) is synthesized according to the literature.^[^ ^60^ ^]^ High‐entropy oxide precursor is prepared with five polyacetylacetone complexes mixed in a stoichiometric ratio and dissolved in methanol at a ratio of 0.65:1 (metal source to methanol) to form the precursor sol. The spinning solution was then prepared by adding 0.3 wt% PEO to this sol. GLTZH fiber aerogels are subsequently prepared using the electrospinning process. The precursor aerogel is collected and arranged on a corundum plate, and covered with corundum pillars and plates of varying heights to obtain GLTZH aerogel of different densities (4.35 ≈ 132.99 mg cm^−3^). The precursor aerogel is heat‐treated in air to obtain a high‐entropy fiber aerogel under specific heating schemes. The temperature is primarily raised to 200 and 750 °C at a heating rate of 1 °C min^−1^ and held for 30 min, respectively. Then, it is continuously elevated up to 1000 °C at a rate of 2 °C min^−1^ and maintained for 2 h.

### Characterization and Measurement

The thermal decomposition process of the precursor from room temperature to 1200 °C is conducted in air using a differential scanning calorimeter with a heating rate of 10 °C min^−1^ (TG/DSC, Netzsch STA 449 F3, Germany). Phase transitions at various treatment temperatures are analyzed using an X‐ray diffractometer by a Cu Kα radiation source with a wavelength of 1.540598 Å (XRD, EMPYREAN, Netherlands). Chemical component evolution from room temperature to 1100 °C is monitored using an infrared spectrometer (FT‐IR; Thermo Fisher Scientific, Germany). The grain size and morphology changes are examined using a scanning electron microscope (SEM, Tescan Mira3, Czech) and a transmission electron microscope (TEM, FEITalosF200X, USA) with the sample prepared by a powder drop‐casting method. The nucleation and grain growth at different temperatures are tracked in situ with a STEM with a heating rate of 10 °C min^−1^ and a holding time of 30 min at each time point. Oxygen vacancies are detected using electron paramagnetic resonance spectroscopy (EPR, Bruker EMXplus‐6/1). The compressive properties are assessed using a universal mechanical testing machine (Shimadzu EZ‐SX, Japan). Thermal conductivity at various temperatures is measured using a thermal conductivity meter (Hot Disk‐hotdisk2500s, Sweden). The infrared images of the samples were recorded using an infrared camera (FLIR T540, USA) with imaging temperatures ranging from 25 to 1500 °C.

### Theoretical Calculations

The CP2K software package was selected for first‐principles calculation based on density functional theory.^[^ ^61^ ^]^ The computational model consisted of a 4×4×4 supercell containing a total of 704 atoms, with the corresponding cell dimensions fixed at 21.034 × 21.034 × 21.034 Å. Brillouin‐zone sampling was restricted to the Γ point due to the substantial size of the supercell. Exchange–correlation interactions were treated within the generalized gradient approximation using the PBE‐SOL functional. Norm‐conserving pseudopotentials were employed to describe the ionic core–valence electron interactions, with a plane‐wave cutoff energy of 600 eV. The electronic self‐consistency and atomic force criteria were converged to tighter than 10^−7^ eV in total energy and 10^−5^ eV Å^−1^ in forces, respectively.

Ab initio molecular dynamics (AIMD) simulations were performed using the same supercell model as described above. Brillouin‐zone sampling was restricted to the Γ point, and exchange–correlation effects were treated within the generalized gradient approximation using the PBE‐SOL functional. The self‐consistent field (SCF) convergence threshold was set to 10^−5^ eV. Canonical ensemble (NVT) simulations were carried out using a velocity‐rescaling thermostat with a relaxation time constant of 100 fs to ensure proper canonical sampling. The time step is set at 2.0 fs, leading to a total of 2000 steps, amounting to a simulation duration of 4 ps.

## Conflict of Interest

The authors declare no conflict of interest.

## Supporting information



Supporting Information

Supplemental Movie 1

Supplemental Movie 2

Supplemental Movie 3

Supplemental Movie 4

Supplemental Movie 5

Supplemental Movie 6

## Data Availability

The data that support the findings of this study are available from the corresponding author upon reasonable request.

## References

[1] Y. An , K. Wan , M. Song , L. Zhao , Ceram. Int. 2024, 50, 4699.

[2] J. Tang , X. Wei , W. Liu , J. Qin , Y. Lv , L. Zhou , S. Wang , X. Long , Y. Lin , J. Liao , ACS Appl. Mater. Interfaces 2025, 17, 38357. 40545617 10.1021/acsami.5c07350

[3] M. Yan , Y. Fu , Y. Pan , X. Cheng , L. Gong , Y. Zhou , H. Ahmed , H. Zhang , Compos. Part B Eng. 2022, 230, 109496.

[4] Z. Deng , Y. Peng , W. W. Qin , B. Liu , G. Zhang , X. Wang , Y. Xie , L. Zhu , D. Xu , Chem. Eng. J. 2023, 475, 146260.

[5] Y. Si , X. Wang , L. Dou , J. Yu , B. Ding , Sci. Adv. 2018, 4, aas8925. 10.1126/sciadv.aas8925 PMC5922795 29719867

[6] L. Song , F. Zhang , Y. Chen , L. Guan , Y. Zhu , M. Chen , H. Wang , B. R. Putra , R. Zhang , B. Fan , Nano Micro Lett 2022, 14, 152. 10.1007/s40820-022-00905-6 PMC9334492 35900619

[7] B. Shi , Y. Qin , Z. Zhou , Y. Chen , Y. Zhang , D. Guo , N. Zhou , L. Wang , B. Xu , Adv. Funct. Mater. 2025, 12480.

[8] Y. Cheng , B. Ma , P. Hu , J. Zhang , D. Hu , J. Wang , Adv. Funct. Mater. 2023, 33, 2309148.

[9] M. Gao , B. Liu , P. Zhao , X. Yi , X. Shen , Y. Xu , J. Sol–gel Sci. Technol. 2019, 91, 514.

[10] J. Guo , S. Fu , Y. Deng , X. Xu , S. Laima , D. Liu , P. Zhang , J. Zhou , H. Zhao , H. Yu , S. Dang , J. Zhang , Y. Zhao , H. Li , X. Duan , Nature 2022, 606, 909. 35768591 10.1038/s41586-022-04784-0 PMC9242853

[11] M. Liu , Y. Kong , J. Tang , B. Zhang , X. Shen , Compos. Part Appl. Sci. Manuf. 2025, 190, 108692.

[12] J. He , X. Li , D. Su , H. Ji , X. Wang , J. Eur. Ceram. Soc. 2016, 36, 1487.

[13] F. Xu , Y. Wang , F. Tang , X. Dai , Z. Zhao , Y. Kong , X. Shen , G. Shao , Carbon 2025, 233, 119854.

[14] C. Jia , L. Li , Y. Liu , B. Fang , H. Ding , J. Song , Y. Liu , K. Xiang , S. Lin , Z. Li , W. Si , B. Li , X. Sheng , D. Wang , X. Wei , H. Wu , Nat. Commun. 2020, 11, 3732. 32709868 10.1038/s41467-020-17533-6 PMC7382455

[15] J. Hou , Y. Liu , C. Cheng , F. Cheng , P. Qin , Y. Miao , W. Ji , X. Wang , J. Alloys Compd. 2024, 996, 174774.

[16] K. Chen , X. Pei , L. Tang , H. Cheng , Z. Li , C. Li , X. Zhang , L. An , J. Eur. Ceram. Soc. 2018, 38, 4161.

[17] M. Jiang , R. Li , B. Li , J. Ni , G. Wang , Z. Zhang , Y. Chen , Y. Hu , D. Wu , J. Chu , H. Wu , J. Li , Sci. Adv. 2025, 11, adw6632. 10.1126/sciadv.adw6632 PMC12533655 41105781

[18] Y. Jiao , J. Dai , Z. Fan , J. Cheng , G. Zheng , L. Grema , J. Zhong , H. Li , D. Wang , Mater. Today 2024, 77, 92.

[19] C. Juan , M. Tsai , C. Tsai , W. Hsu , C. Lin , S. Chen , S. Lin , J. Yeh , Mater. Lett. 2016, 184, 200.

[20] Z. Zhao , H. Xiang , F.‐Z. Dai , Z. Peng , Y. Zhou , J. Mater. Sci. Technol. 2019, 35, 2647.

[21] P. Zhang , L. Gong , Z. Lou , J. Xu , S. Cao , J. Zhu , H. Yan , F. Gao , J. Alloys Compd. 2022, 898, 162858.

[22] C. Gao , J. Zhu , S. Ye , M. Li , H. Wang , J. He , J. Eur. Ceram. Soc. 2025, 45, 116878.

[23] L. Su , H. Huyan , A. Sarkar , W. Gao , X. Yan , C. Addiego , R. Kruk , H. Hahn , X. Pan , Nat. Commun. 2022, 13, 2358. 35487934 10.1038/s41467-022-30018-y PMC9055071

[24] C. A. Morris , M. L. Anderson , R. M. Stroud , C. I. Merzbacher , D. R. Rolison , Science 1999, 284, 622. 10213681 10.1126/science.284.5414.622

[25] L. Su , M. Niu , D. Lu , Z. Cai , M. Li , H. Wang , J. Mater. Sci. Technol. 2021, 75, 1.

[26] L. Li , Y. Zhou , Y. Gao , X. Feng , F. Zhang , W. Li , B. Zhu , Z. Tian , P. Fan , M. Zhong , H. Niu , S. Zhao , X. Wei , J. Zhu , H. Wu , Nat. Commun. 2023, 14, 5410. 37670012 10.1038/s41467-023-41087-y PMC10480443

[27] M. Wei , J. Xu , R. Yang , J. Zhu , X. Meng , J. Yang , F. Gao , J. Am. Ceram. Soc. 2022, 105, 4449.

[28] H. Wang , J. Xu , M. Wei , X. Feng , J. Wu , P. Zhang , F. Gao , J. Am. Ceram. Soc. 2024, 107, 3857.

[29] C. Li , D. Jiang , H. Liang , B. Huo , C. Liu , W. Yang , J. Liu , Adv. Funct. Mater. 2018, 28, 1704674.

[30] W. Zhu , Y. Zhuang , J. Weng , Q. Huang , G. Lai , L. Li , M. Chen , K. Xia , Z. Lu , M. Wu , Z. Zou , Adv. Mater. 2024, 2407138. 10.1002/adma.202407138 38887139

[31] J. Yue , M. Qin , H. Yu , Q. He , W. Feng , Adv. Funct. Mater. 2025, 2508319.

[32] S. Shang , J. Wang , M. Yuan , Q. You , Z. Song , W. Liu , X. Ye , J. Yang , S. Cui , Mater. Charact. 2024, 217, 114392.

[33] J. Wang , W. Wang , X. Liu , S. Shang , Y. Chen , L. Shi , S. Cui , Ceram. Int. 2024, 50, 1795.

[34] H.‐N.‐R. Jung , V. G. Parale , H. Choi , J. Kim , W. Lee , S.‐H. Kim , W. K. Jung , H.‐H. Park , J. Alloys Compd. 2024, 980, 173561.

[35] Z. Wu , X. Cheng , L. Zhang , J. Li , C. Yang , Ceram. Int. 2018, 44, 14947.

[36] E. Bonnet , J. C. Grenier , J. M. Bassat , A. Jacob , B. Delatouche , S. Bourdais , J. Eur. Ceram. Soc. 2021, 41, 4505.

[37] Z. Teng , Y. Tan , S. Zeng , Y. Meng , C. Chen , X. Han , H. Zhang , J. Eur. Ceram. Soc. 2021, 41, 3614.

[38] M. R. Chellali , A. Sarkar , S. H. Nandam , S. S. Bhattacharya , B. Breitung , H. Hahn , L. Velasco , Scr. Mater. 2019, 166, 58.

[39] J. Liu , A. Wang , P. Gao , R. Bai , J. Liu , B. Du , C. Fang , J. Am. Ceram. Soc. 2024, 107, 1361.

[40] S. Liu , C. Dun , Q. Jiang , Z. Xuan , F. Yang , J. Guo , J. J. Urban , M. T. Swihart , Nat. Commun. 2024, 15, 1167. 38326434 10.1038/s41467-024-45413-w PMC10850329

[41] S. Zhang , J. Xu , C. Lu , R. Ouyang , J. Ma , X. Zhong , X. Fang , X. Xu , X. Wang , J. Am. Ceram. Soc. 2024, 107, 3475.

[42] Y. Xie , Y. Peng , D. Ma , W. Liu , Z. Deng , L. Zhu , G. Zhang , X. Wang , J. Alloys Compd. 2021, 876, 159978.

[43] C. P R , T. T. John , J. Lumin. 2017, 185, 212.

[44] A. L. Patterson , Phys. Rev. 1939, 56, 978.

[45] L. Su , X. Chen , L. Xu , T. Eldred , J. Smith , C. DellaRova , H. Wang , W. Gao , ACS Nano 2022, 16, 21397. 36454037 10.1021/acsnano.2c09760

[46] Y. Wu , F. Chen , W. Han , T. Zhao , Ceram. Int. 2020, 46, 22102.

[47] M. Cernea , A. Manea , D. Piazza , C. Galassi , E. Vasile , J. Am. Ceram. Soc. 2007, 90, 1728.

[48] Z. Wang , T. Zhou , X. Yang , Y. Liu , Q. Wen , Z. Yu , Materials 2024, 17, 5294. 39517568 10.3390/ma17215294 PMC11547591

[49] H. Yang , S. Klemm , J. Müller , M. F. Bekheet , A. Gurlo , D. A. H. Hanaor , J. Eur. Ceram. Soc. 2023, 43, 4233.

[50] L. Lu , P. Du , T. Jiang , T. Zhou , Q. Wen , Y. Wang , Y. Zeng , X. Xiong , J. Eur. Ceram. Soc. 2025, 45, 116885.

[51] M. J. Hÿtch , E. Snoeck , R. Kilaas , Ultramicroscopy 1998, 74, 131.

[52] K. Kang , Y. Liu , X. Liu , C. Wang , M. Wei , Chem. Eng. J. 2025, 507, 159463.

[53] C. Lee , Y. Chou , G. Kim , M. C. Gao , K. An , J. Brechtl , C. Zhang , W. Chen , J. D. Poplawsky , G. Song , Y. Ren , Y. Chou , P. K. Liaw , Adv. Mater. 2020, 32, 2004029. 10.1002/adma.202004029 33135322

[54] R. Yao , Z. Yao , J. Zhou , J. Appl. Polym. Sci. 2018, 135, 45953.

[55] R. Zhang , Q. Qu , B. Han , B. Wang , Mater. Lett. 2016, 175, 219.

[56] Y. Liu , Z. Zhao , Y. Kong , C. Chu , J. Tang , J. Ren , X. Shen , Ceram. Int. 2022, 48, 27486.

[57] T. Xue , Y. Yu , Z. Fu , Q. Wang , Z. Hu , W. Fan , T. Liu , Sci. Technol. 2023, 242, 110196.

[58] J. Feng , C. Zhang , J. Feng , Y. Jiang , N. Zhao , ACS Appl. Mater. Interfaces 2011, 3, 4796. 22047011 10.1021/am201287a

[59] H. Wang , X. Zhang , N. Wang , Y. Li , X. Feng , Y. Huang , C. Zhao , Z. Liu , M. Fang , G. Ou , H. Gao , X. Li , H. Wu , Sci. Adv. 2017, 3, 1603170. 10.1126/sciadv.1603170 PMC5457032 28630915

[60] N. Cai , X. Wang , D. Guo , L. Zhu , G. Zhang , X. Duan , D. Xu , Mater. Lett. 2015, 153, 191.

[61] T. D. Kühne , M. Iannuzzi , M. Del Ben , V. V. Rybkin , P. Seewald , F. Stein , T. Laino , R. Z. Khaliullin , O. Schütt , F. Schiffmann , D. Golze , J. Wilhelm , S. Chulkov , M. H. Bani‐Hashemian , V. Weber , U. Borštnik , M. Taillefumier , A. S. Jakobovits , A. Lazzaro , H. Pabst , T. Müller , R. Schade , M. Guidon , S. Andermatt , N. Holmberg , G. K. Schenter , A. Hehn , A. Bussy , F. Belleflamme , G. Tabacchi , et al., J. Chem. Phys. 2020, 152, 194103. 33687235 10.1063/5.0007045

