# Supplementary material for: Superelastic High‐Entropy Oxide Ceramic Aerogels for Thermal Superinsulation and Sealing at Extreme Conditions
Source: Adv Sci (Weinh). 2025 Dec 3;13(11):e16840. doi: 10.1002/advs.202516840 (PMC12931259; doi:10.1002/advs.202516840)
Supplement: Supplementary file 1 — Supporting Information [file ADVS-13-e16840-s005.docx]

Supplementary Information

**Superelastic High-entropy Oxide Ceramic Aerogels for Thermal Superinsulation at Extreme Conditions**

*Xiaoke Jiang#, Tao Du#, Hengzhong Fan*, Junhong Liu, Yunfeng Su, Peng He, Hongxaing Chen, Litian Hu, Yongsheng Zhang*, Qiangqiang Zhang**

X. K. Jiang, H. Z. Fan, Y. F. Su, L. T. Hu, Y. S. Zhang

State Key Laboratory of Solid Lubrication, Lanzhou Institute of Chemical Physics, Chinese Academy of Sciences, Lanzhou 730000, China

E-mail: hzhfan@licp.cas.cn, zhysh@licp.cas.cn

X. K. Jiang, H. Z. Fan, Y. F. Su, L. T. Hu, Y. S. Zhang

Center of Materials Science and Optoelectronics Engineering, University of Chinese Academy of Sciences, Beijing 100049, China

T. Du

Key Lab of Smart Prevention and Mitigation of Civil Engineering Disasters of the Ministry of Industry and Information Technology, Harbin Institute of Technology, Harbin, 150090 China

1. H Liu, P. He, Q. Q. Zhang

College of Civil Engineering and Mechanics, Lanzhou University, Lanzhou 730000, China

Key Laboratory of Mechanics on Disaster and Environment in Western China (Lanzhou University), The Ministry of Education of China, Lanzhou 730000, China

1. mail: zhangqq@lzu.edu.cn

H. X. Chen

School of Materials Science and Engineering, Fujian University of Technology, Fuzhou, 350118, PR China

**Table of Contents**

**S1:** The element design of high entropy oxide

**S2:** Structural characterization of the ZHTLG oxide.

**S3:** Phase identification of ZHTLG oxide.

**S4:** In-situ evolution process of ZHTLG oxide.

**S5:** Thermal stability comparison of different oxide ceramic fibers.

**S6:** Detailed description of AIMD computational models.

**S7:** References

**S1: The element design of high entropy oxide**

The elemental selection in this work is guided by a clear synergistic design strategy, which considers both the physical properties of individual components and key factors affecting single-phase formation, such as electronegativity and ionic size difference. This rationale leads to the selection of five cations: Zr, Hf, Ti, Lu, and Gd. Their relevant physical properties are summarized in **Table S1**.

**Table S1**. Key physicochemical parameters for element selection

| Element | Charge | Coordination Number | Melting Point (℃) | electronegativity | ionic radius |
| --- | --- | --- | --- | --- | --- |
| **Lu** | **3+** | **6** | **2487** | **1.27** | **0.977** |
| **Gd** | **3+** | **6** | **2350** | **1.20** | **1.053** |
| **Zr** | **4+** | **8** | **2715** | **1.33** | **0.84** |
| **Hf** | **4+** | **8** | **2810** | **1.30** | **0.83** |
| **Ti** | **4+** | **8** | **1875** | **1.54** | **0.745** |
| La | 3+ | 6 | 2315 | 1.10 | 1.032 |
| Y | 3+ | 6 | 2458 | 1.22 | 0.9 |
| Sm | 3+ | 6 | 2350 | 1.13 | 0.958 |
| Ce | 4+ | 8 | 2600 | 1.12 | 0.97 |

ZrO_2_ and HfO_2_, widely used in thermal barrier coatings, form the thermally stable backbone of the material system due to their high melting points and low thermal conductivity^[1,2]^. However, ZrO_2_ and HfO_2_ suffer from phase transitions at high temperatures and possess limited capability to regulate radiative heat transfer^[3]^. To address this, TiO_2_ is introduced to enhance the radiative shielding performance at high temperatures^[4]^. Since the incorporation of TiO_2_ may reduce the overall melting point, high-melting-point rare-earth oxides, La, Y, Sm, Lu, Gd and Ce are further doped to increase the service temperature while simultaneously stabilizing the phase structure.

The formation of a single-phase solid solution is influenced by the degree of atomic size disorder and the difference in electronegativity among constituent elements. According to the Hume–Rothery rules, a smaller difference in ionic radii favors the formation of continuous solid solutions, while a larger variance in electronegativity tends to inhibit solid solubility^[5,6]^.

**Table S2.** The size disorder degree and electronegativity variance of different samples

| Sample | Size disorder degree (*δ*) / % | Electronegativity variance ($\text{σ}_{\text{x}}^{\text{2}}$) / % |
| --- | --- | --- |
| ZrHfTiCeSm | 9.75 | 2.37 |
| ZrHfTiGdLa | 13.56 | 2.16 |
| ZrHfTiLaY | 10.93 | 2.10 |
| ZrHfTiLuGd | 12.45 | 1.31 |

The size disorder degree (*δ*) is defined by the following expression:

$\delta=\sqrt{\sum_{i=1}^{n} x_{i}{(1-\frac{r_{i}}{r})}^{2}}$ (**1**)

where $r_{i}$ is the ionic radius of each metal element in the system, $r$ is the average ionic radius of all metal elements, $x_{i}$ is the mole fraction of each metal element, and $n$ is the total number of metal elements in the system.

The electronegativity variance ($\sigma_{x}^{2}$) is given by:

$\sigma_{x}^{2}=\sum_{i=1}^{n} x_{i}{(\chi_{i}-\chi)}^{2}$ (**2**)

where $\chi_{i}$ represents the electronegativity of each metal element, and $\chi$ is the average electronegativity of all metal elements in the system.

According to **Table S2**, the size disorder degree of all components is less than 15%, which satisfies the Hume-Rothery rule for forming a single-phase solid solution. Moreover, the electronegativity variances are all relatively small, among which ZHTLG has the smallest electronegativity variance.


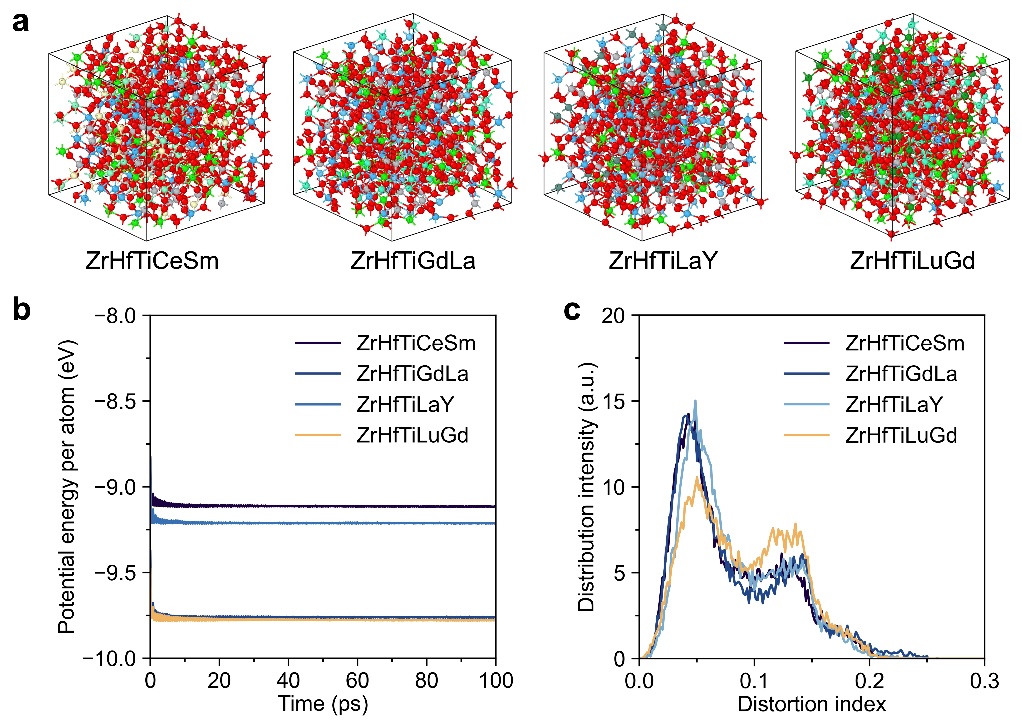


**Figure S1**. (a) Atomic snapshots of the equilibrated structures of HEC with different compositions. (b) Evolution of potential energy of HEC during the equilibration process under 300 K and ambient pressure. (c) Distortion index of the Ti–O polyhedra in HEC with different compositions.

The selection of Gd, Hf, Lu, Ti, and Zr as cations is based on both thermodynamic and structural considerations that ensure the formation of a single-phase high entropy oxide (HEO) with a desirable functional stability. The combination of trivalent (Gd^3+^, Lu^3+^) and tetravalent (Hf^4+^, Ti^4+^, Zr^4+^) cations maintains overall charge neutrality (O^2-^ anions) while maximizing cationic configurational entropy, which enables to stabilize a single-phase solid solution at high temperature. The ionic radii of these cations span a moderate range (e.g., Gd^3+^≈1.053 Å; Lu^3+^≈0.977 Å; Ti^4+^≈0.745 Å; Zr^4+^≈0.84 Å; Hf^4+^≈0.83 Å), promoting local lattice distortion that enhances phonon scattering and potentially improves thermal stability and mechanical robustness.

Given these considerations, we selecte the elements of Zr, Hf, Ti, Lu, and Gd for our HEC composition. In comparison, we also consider the compositions ZrHfTiGdLa, ZrHfTiLaY, and ZrHfTiCeSm, which involve substituting the trivalent cations (**Figure S1**a). During the equilibration process of these four compositions, we observe that the selected ZrHfTiLuGd composition exhibits the lowest potential energy over time, indicating an adequately long equilibration period and the greatest thermodynamic stability compared to the other three phases. The mixed coordination states in the various HEC samples are evident, as demonstrated by the bimodal distribution of the distortion index for the Ti–O polyhedra. However, the Ti–O polyhedra in the selected ZrHfTiLuGd composition exhibit the most disordered structure, suggesting that the HEC of ZrHfTiLuGd combines both thermal stability and high configurational entropy.

**S2: Structural characterization of the ZHTLG oxide**


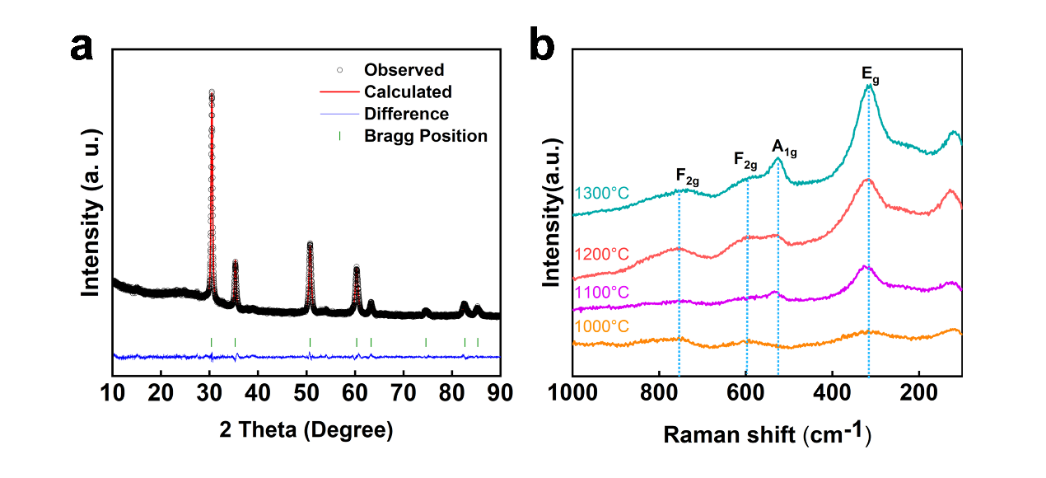


**Figure S2.** (a) XRD refinement of the ZHTLG oxide annealing at 1000 ℃. (b) Raman spectroscopy of the ZHTLG oxide annealed at different temperatures.

**Figure S2**a indicates that the ZHTLG oxide exhibits a single disordered fluorite structure. **Figure S2**b shows that a disordered fluorite structure is presented below 1100 °C. According to group theory, four Raman vibration modes belonging to the pyrochlore sub-phase are observed, indicating that with the increase of sintering temperature, ZHTLG gradually transforms from the disordered fluorite phase to the ordered fluorite phase.

**Table S3.** Retrieved refinement of the ZHTLG material.

| Sample | ZHTLG |
| --- | --- |
| Space group | *F*m 3m |
| $\chi^{2}$ | 2.98 |
| *R*_wp_ | 3.85% |
| a=b=c (Å) | 5.08419 |
| $\alpha=\beta=\gamma$ (°) | 90 |
| Gd | 0.00000(0), 0.00000(0), 0.00000(0) |
| Lu | 0.00000(0), 0.00000(0), 0.00000(0) |
| Zr | 0.00000(0), 0.00000(0), 0.25000(0) |
| Ti | 0.00000(0), 0.00000(0), 0.00000(0) |
| Hf | 0.00000(0), 0.00000(0), 0.00000(0) |
| O | 0.25000(0), 0.25000(0), 0.25000(0) |

As listed in **Table S3**, the analysis and fitting using the Retrieved refinement method determine the unit cell parameters and atomic positions of the ZHTLG. An R_wp_ value of less than 10% indicates that the results are reliable.

**Table S4.** Interplanar distance calculated from the XRD pattern

| Miller indices | 2*θ* (°) | Interplanar distance (nm) |
| --- | --- | --- |
| 111 | 30.427 | 0.294 |
| 200 | 35.277 | 0.254 |
| 220 | 50.747 | 0.180 |
| 311 | 60.329 | 0.153 |
| 222 | 63.313 | 0.147 |
| 400 | 74.605 | 0.127 |
| 331 | 82.660 | 0.117 |
| 420 | 85.305 | 0.137 |
| 422 | 95.842 | 0.104 |

As listed in **Table S4**, the analysis and calculation using the Rietveld refinement method determine the structural parameters of the ZHTLG.

**S2: Phase identification of ZHTLG oxide**


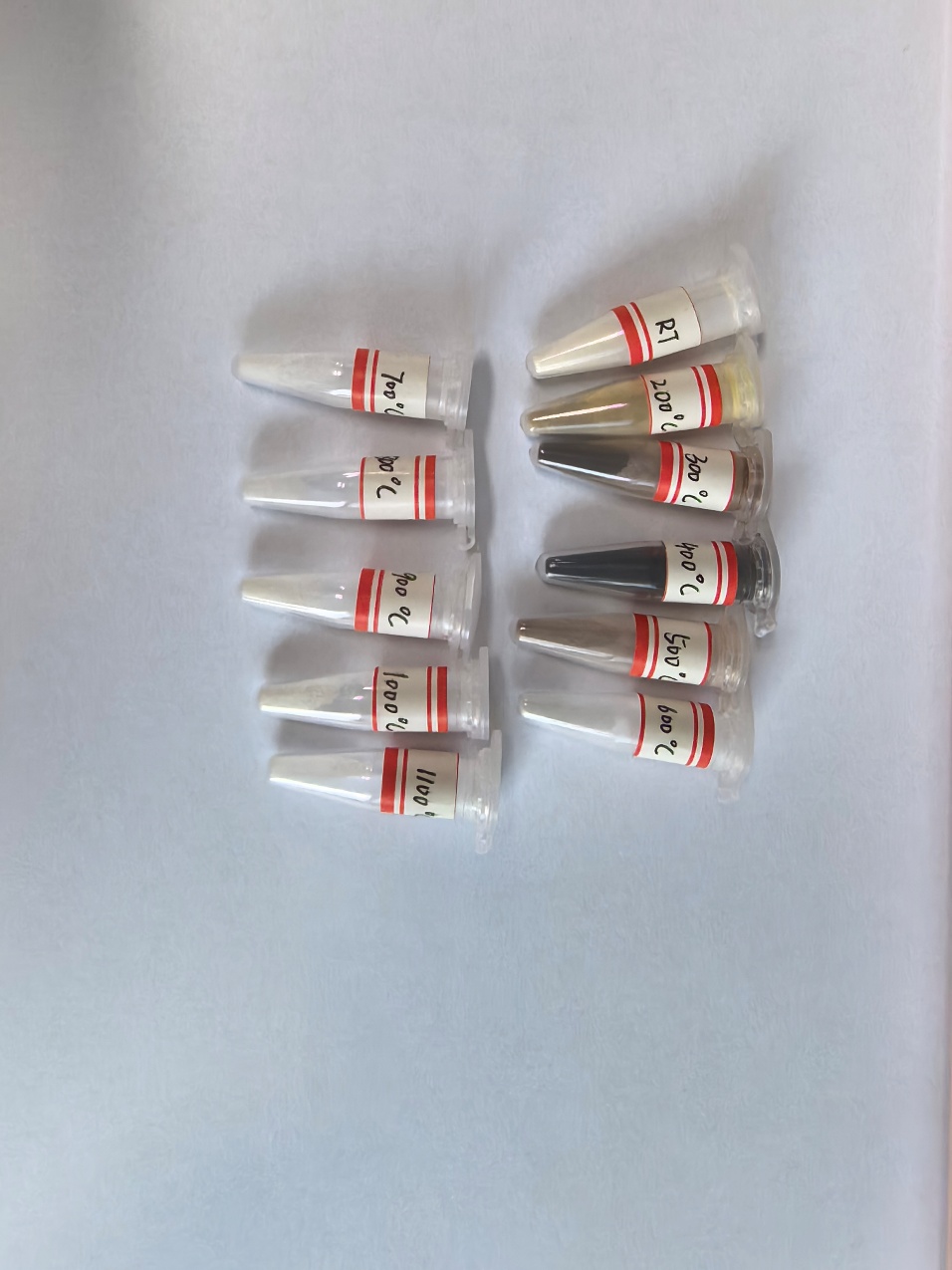


**Figure S3.** Color change of ZHTLG precursors after treated under different temperatures from 200 to 1100°C.

**Figure S3** demonstrates that the precursor exhibits a color change from yellow to black and then to white at different temperatures, which correspond to the carbonization and oxidation of the precursor, respectively.


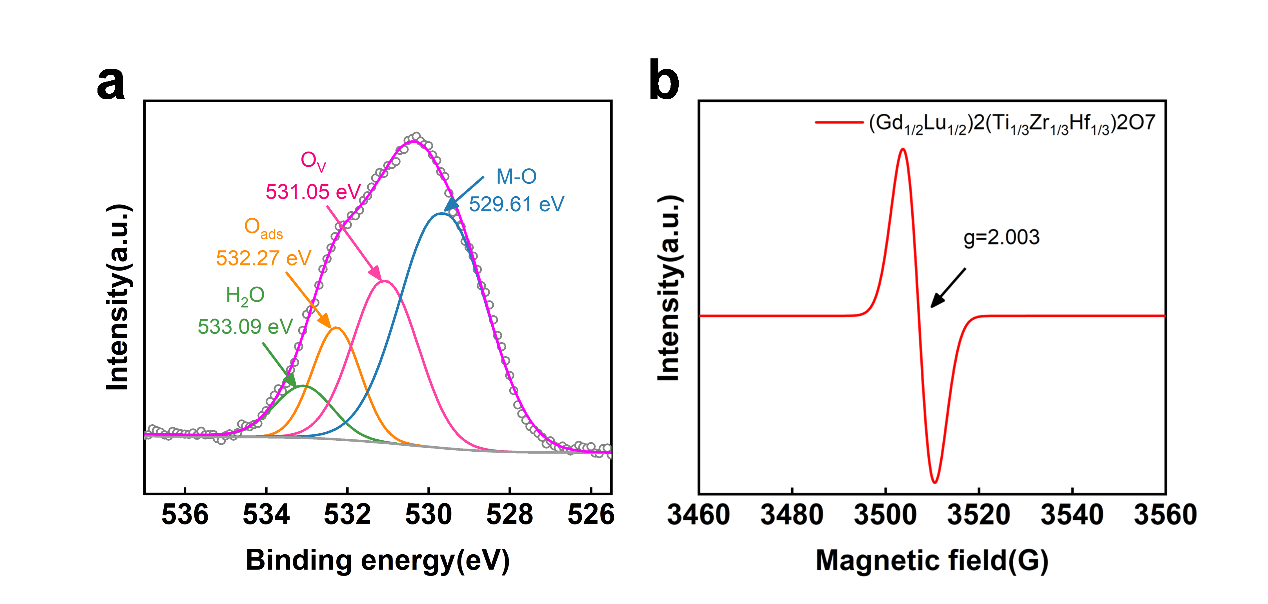


**Figure S4.** (a) O1s spectra of ZHTLG after sintering at 1000 °C. (b) ZHTLG EPR curve.

The O1s spectrum of **Figure S4**a is deconvoluted into four peaks centered at binding energies of 529.61 eV, 531.05 eV, 532.27 eV, and 533.09 eV, respectively. The peak at 529.61 eV is attributed to lattice oxygen (M-O), the peak at 531.05 eV corresponds to oxygen vacancies (Ov), the peak at 532.27 eV arises from surface-adsorbed oxygen species (e.g., O₂⁻, O⁻), and the peak at 533.09 eV is associated with chemisorbed water or hydroxyl groups. The g-factor plot in **Figure S4**b is found to be 2.003, which proves that oxygen vacancies do exist in the sample.

**
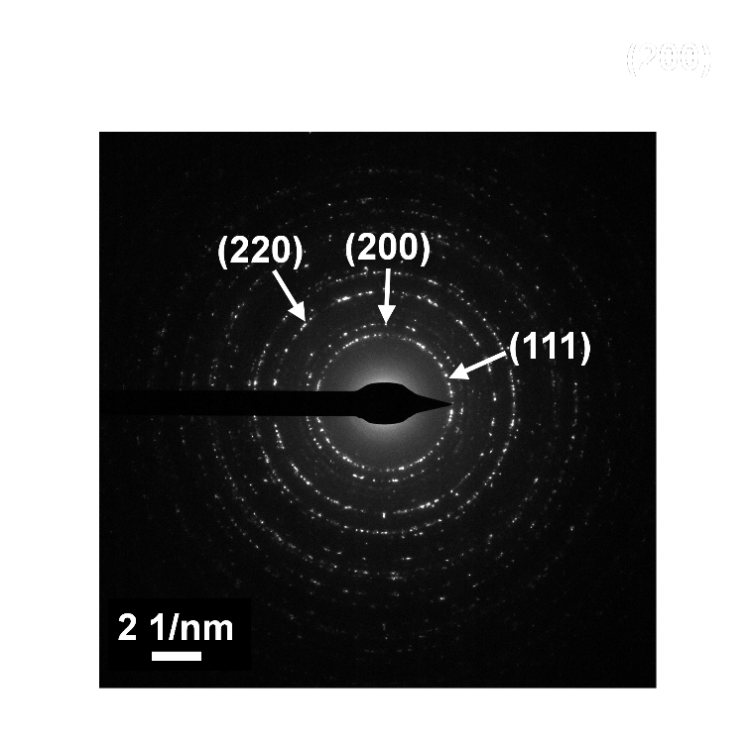
**

**Figure S5.** SAED patterns of ZHTLG oxide annealed at 1100 °C.

**S3: In-situ evolution process of ZHTLG oxide**


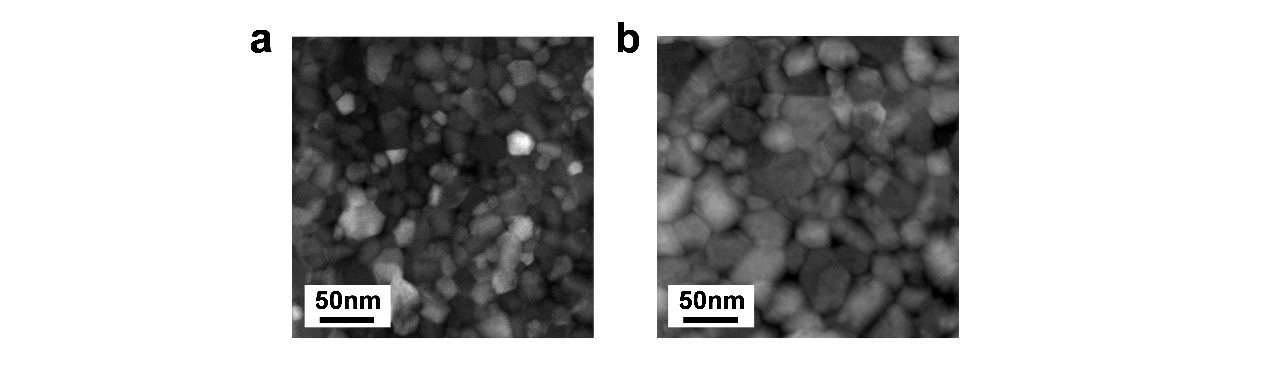


**Figure S**6. Grain size images after annealing at (a) 1000 ℃ and (b) 1100 ℃


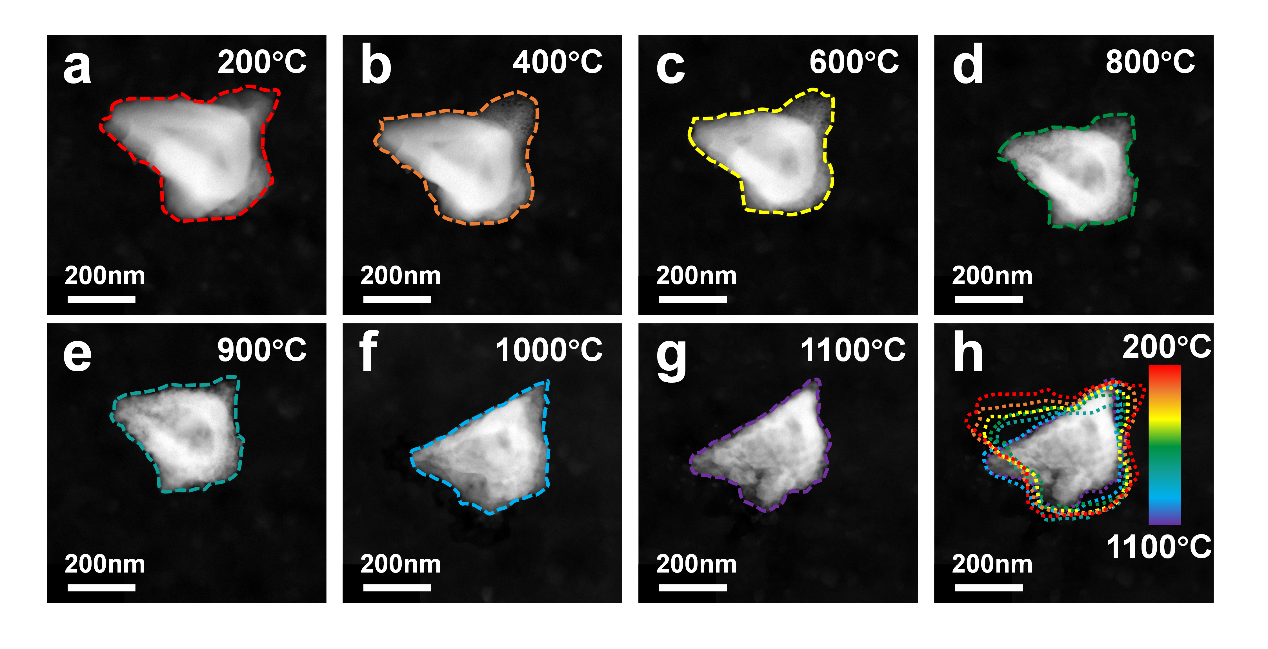


**Figure S7.** HAADF-STEM images of ZHTLG precursor evolution at (a) 200°C. (b) 400°C. (c) 600°C. (d) 800°C. (e) 900°C. (f) 1000°C and (g) 1100°C. (h) The contour map of particle morphology evolution at different annealing stages.

**Figure S7** demonstrates that the volume of ZHTLG precursor particles gradually decrease with increasing temperature, which is consistent with the transformation law from polymer to inorganic phase. Meanwhile, the pores generated by polymer decomposition at lower temperature stages disappear with the annealing progression at higher temperatures.


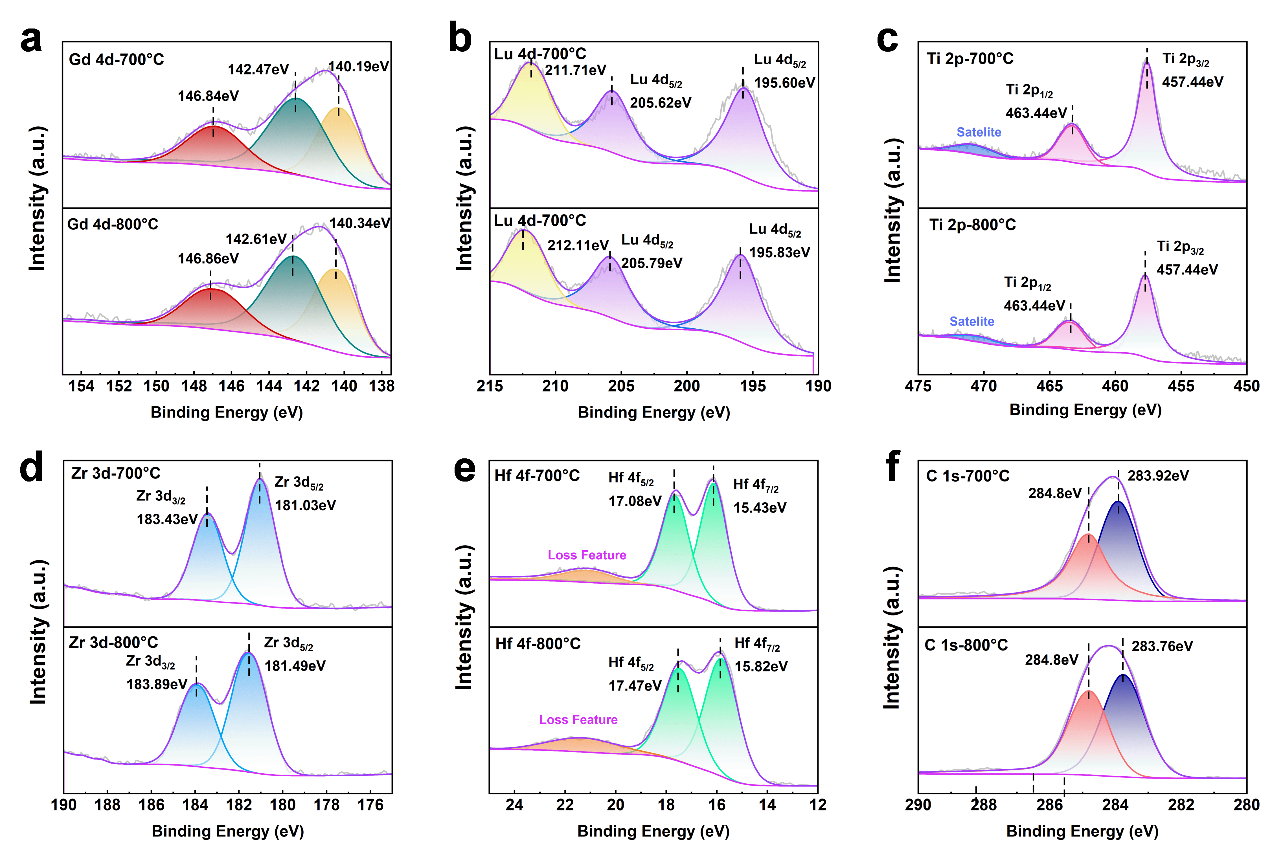


**Figure S8.** XPS Spectra of (a) Gd 4d, (b) Lu 4d, (c) Zr 3d, (d) Hf 4f, (e) Ti 2p, and (f) C 1s at 700°C and 800°C, respectively.


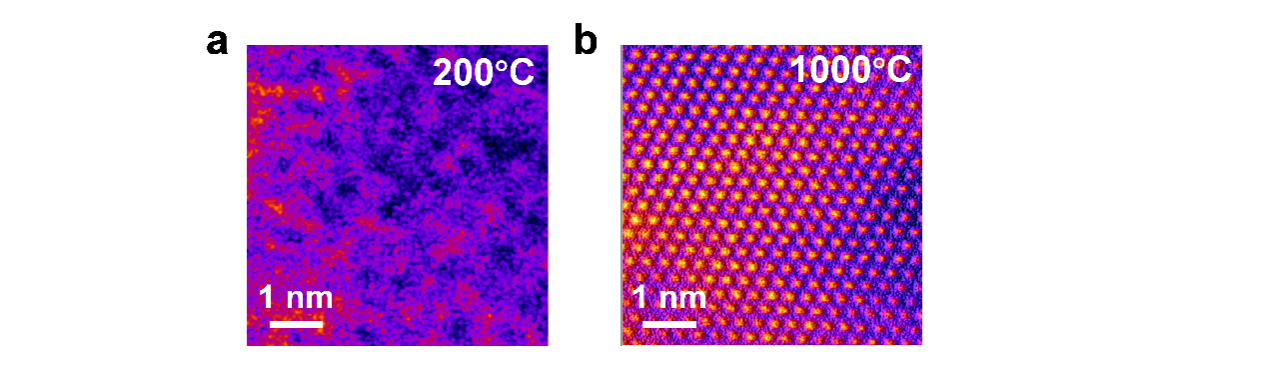


**Figure S9.** Atomic-scale evidence of HEC formation during polymer-to-ceramic conversion at different temperatures of 200 ℃.


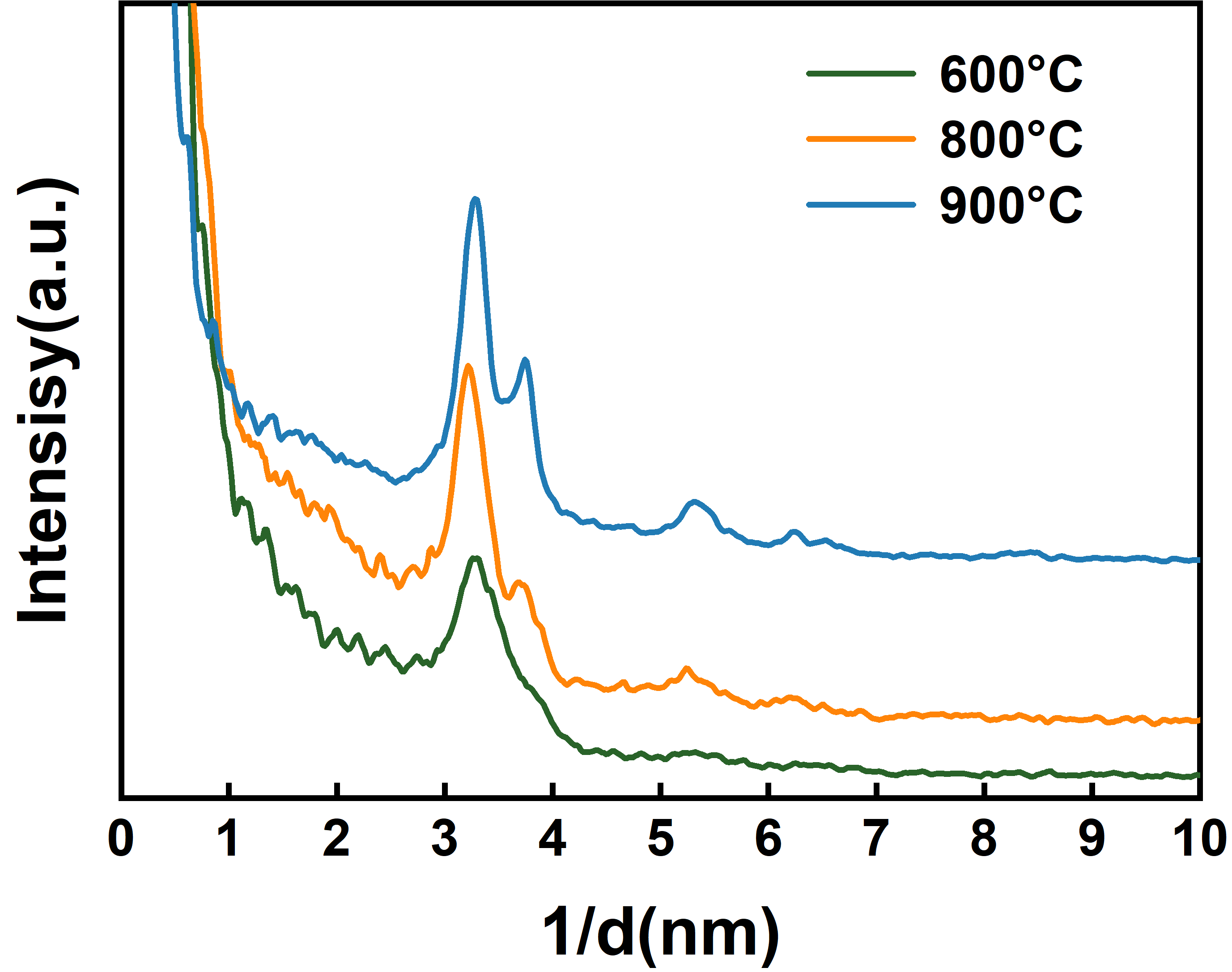


**Figure S10.** Power spectra changes calculated by FFT.


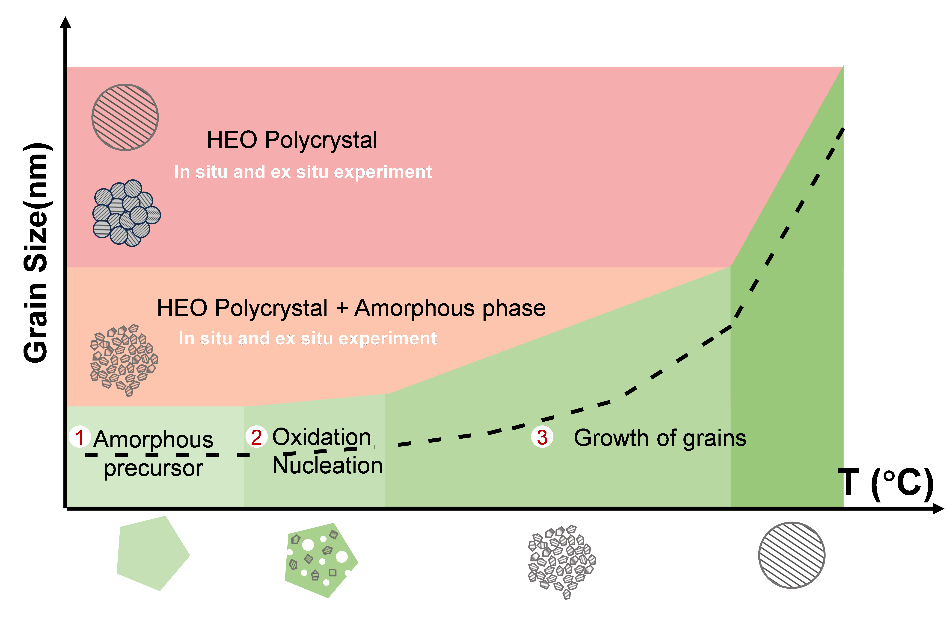


**Figure S11.** Schematic of the ZHTLGformation mechanism.


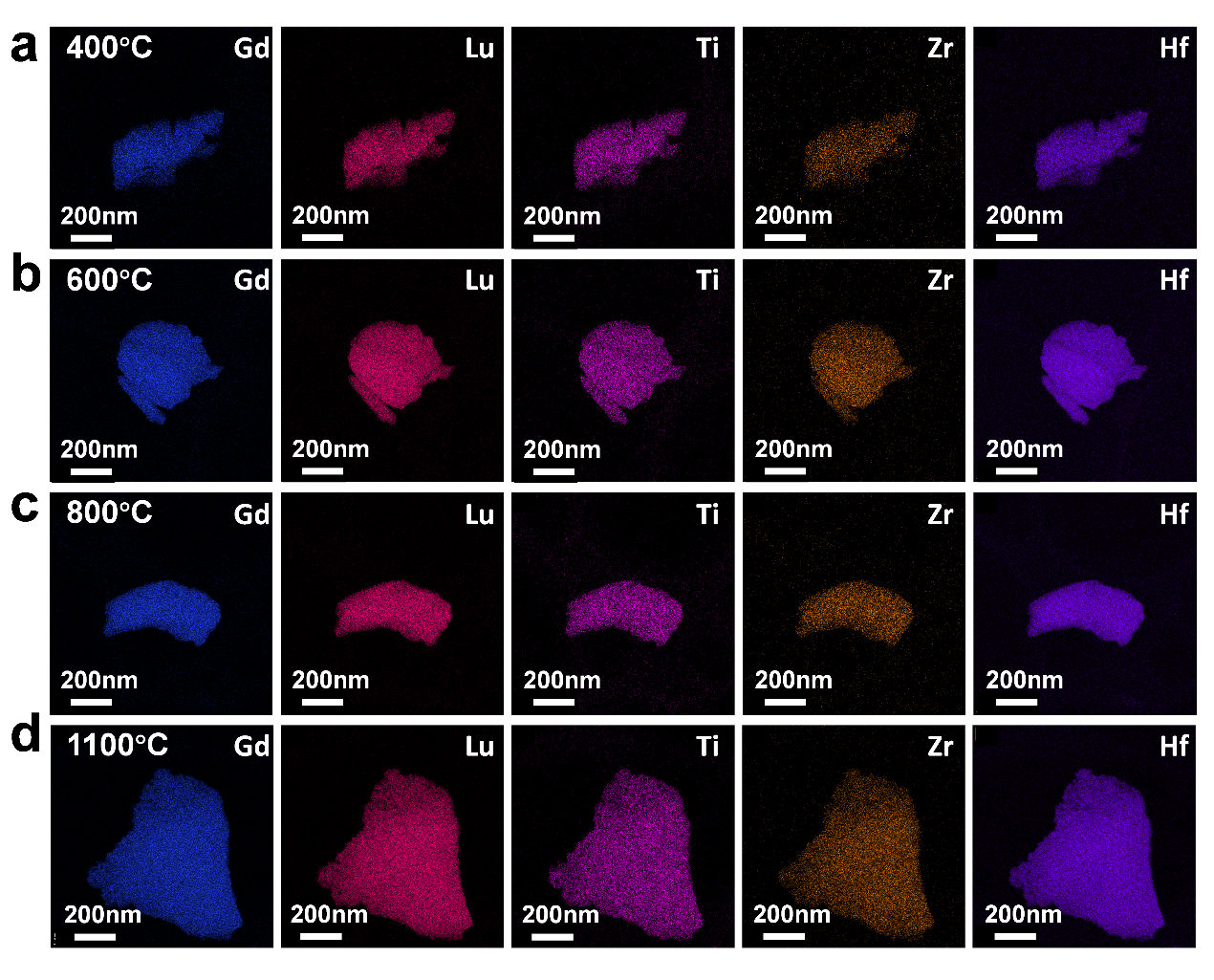


**Figure S12.** EDS mapping of Gd, Lu, Ti, Hf and Zr in ZHTLG at (a) 400 °C. (b) 600 °C. (c) 800 °C. (d)1100 °C.

**S4: Thermal stability comparison of different oxide ceramic fibers**


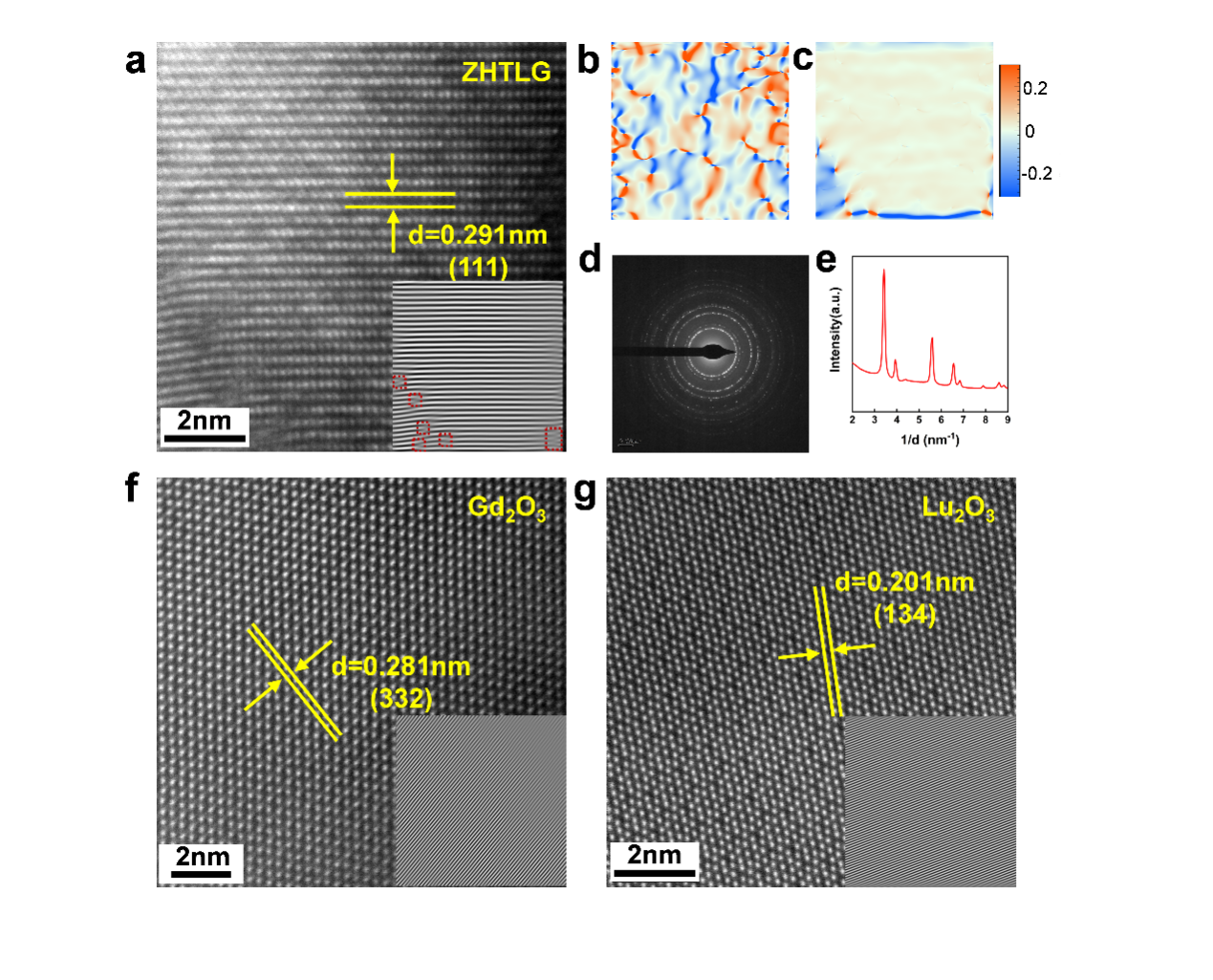


**Figure S12.** Lattice Distortion in ZHTLG. (a) HRTEM image of ZHTLG with IFFT image. (b)-(c) GPA mapping along the xx and yy directions, respectively. (d) SAED patterns of ZHTLG oxide annealed at 1000 °C (e) Power spectra changes calculated by (e). (f) HRTEM image of Gd_2_O_3_ with IFFT. (g) HRTEM image of Lu_2_O_3_ with IFFT.

**Table S5.** ZHTLG and the corresponding oxide crystal structures and unit cell parameters

| Oxide | Crystalline | | *a* | *b* | *c* | PDF-# |
| --- | --- | --- | --- | --- | --- | --- |
| ZrO_2_ | | Monoclinic | 5.145 | 5.210 | 5.311 | 86-1451 |
|  |  | Tetragonal | 3.592 | 3.592 | 5.184 | 80-0965 |
|  |  | Cubic | 5.135 | 5.135 | 5.135 | 89-9069 |
| HfO_2_ | | Monoclinic | 5.118 | 5.186 | 5.284 | 78-0050 |
|  |  | Cubic | 5.115 | 5.115 | 5.115 | 70-2831 |
| TiO_2_ | | Tetragonal (Anatase) | 3.784 | 3.784 | 9.515 | 71-1166 |
|  |  | Tetragonal (Rutile) | 4.594 | 4.594 | 2.959 | 73-2224 |
| Gd_2_O_3_ | | Cubic | 10.809 | 10.809 | 10.809 | 86-2477 |
| Lu_2_O_3_ | | Cubic | 11.258 | 11.258 | 11.258 | 86-2475 |
| **ZHTLG** | | **Cubic** | **5.084** | **5.084** | **5.084** | **-** |


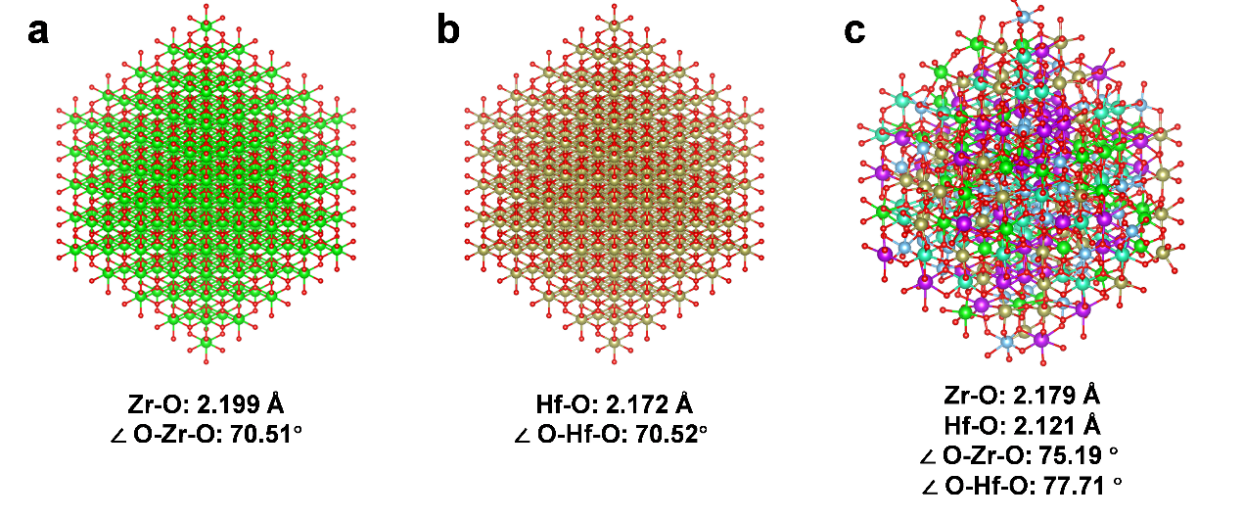


**Figure S14.** The optimized structures of (a) ZrO_2_, (b) HfO_2_ and (c) ZHTLG models, marked with the ∠TM─O─TM bond angles and TM─O bond lengths.

To quantify the changes in bond lengths and bond angles induced by lattice distortion, 4×4×4 supercell models of ZrO_2_, HfO_2_, and ZHTLG are constructed. The random substitution method is adopted to ensure the randomness of metal cations during cell construction. **Figure S14** presents the optimized cell models of the single-component and quinary high-entropy phases, with the changes in bond lengths and bond angles labeled. It can be clearly observed that in ZHTLG, the TM-O bond lengths and ∠TM-O-TM angles have undergone significant variations, accompanied by changes in unit cell volume and shape—i.e., lattice distortion.


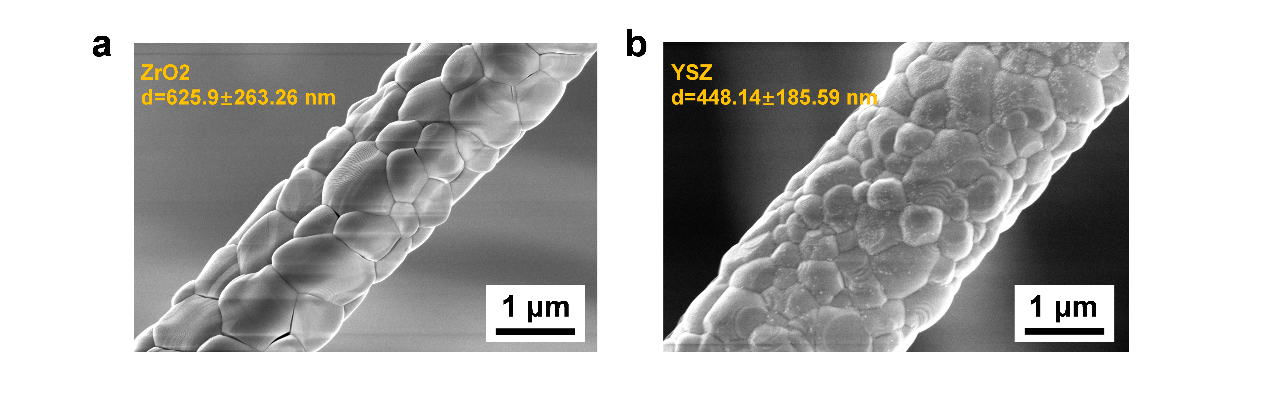


**Figure S15**. The grains size changes after thermal exposure at 1400 ℃ for (a) ZrO_2_ (b) YSZ.

**Table S6.** Melting points of corresponding oxides

| Oxide | Charge | Coordination Number | Melting Point (℃) |
| --- | --- | --- | --- |
| Lu_2_O_3_ | 3+ | 6 | 2487 |
| Gd_2_O_3_ | 3+ | 6 | 2350 |
| ZrO_2_ | 4+ | 8 | 2715 |
| HfO_2_ | 4+ | 8 | 2810 |
| TiO_2_ | 4+ | 8 | 1875 |


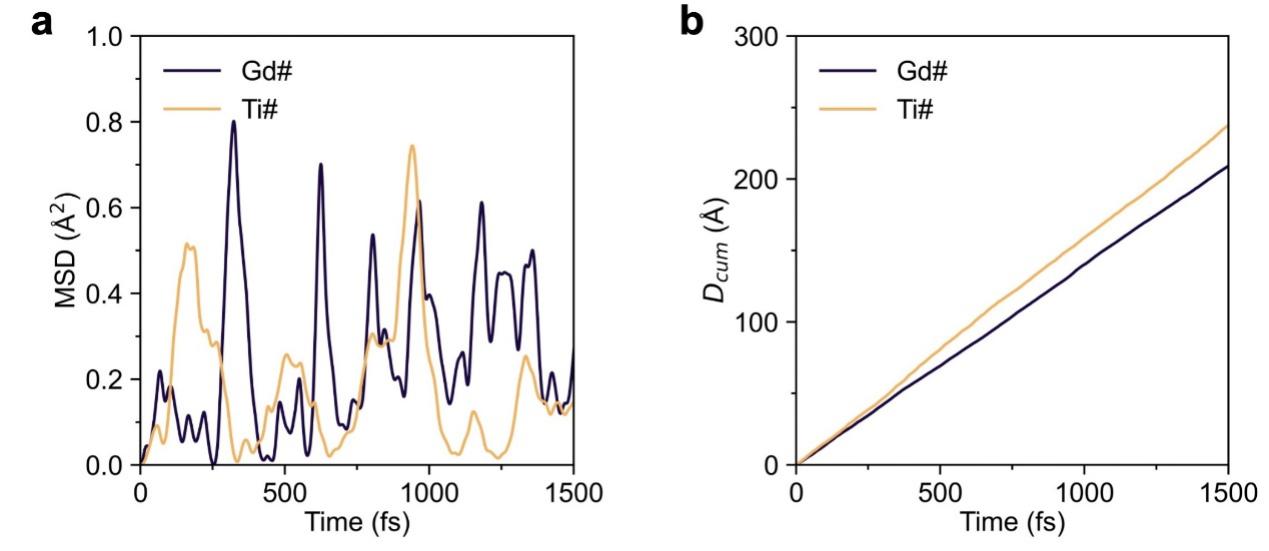


**Figure S16.** (a) Mean squared displacements (MSD). (b) Cumulative non-affine square displacements of selected Gd and Ti atoms during the AIMD simulation of 1273 K.


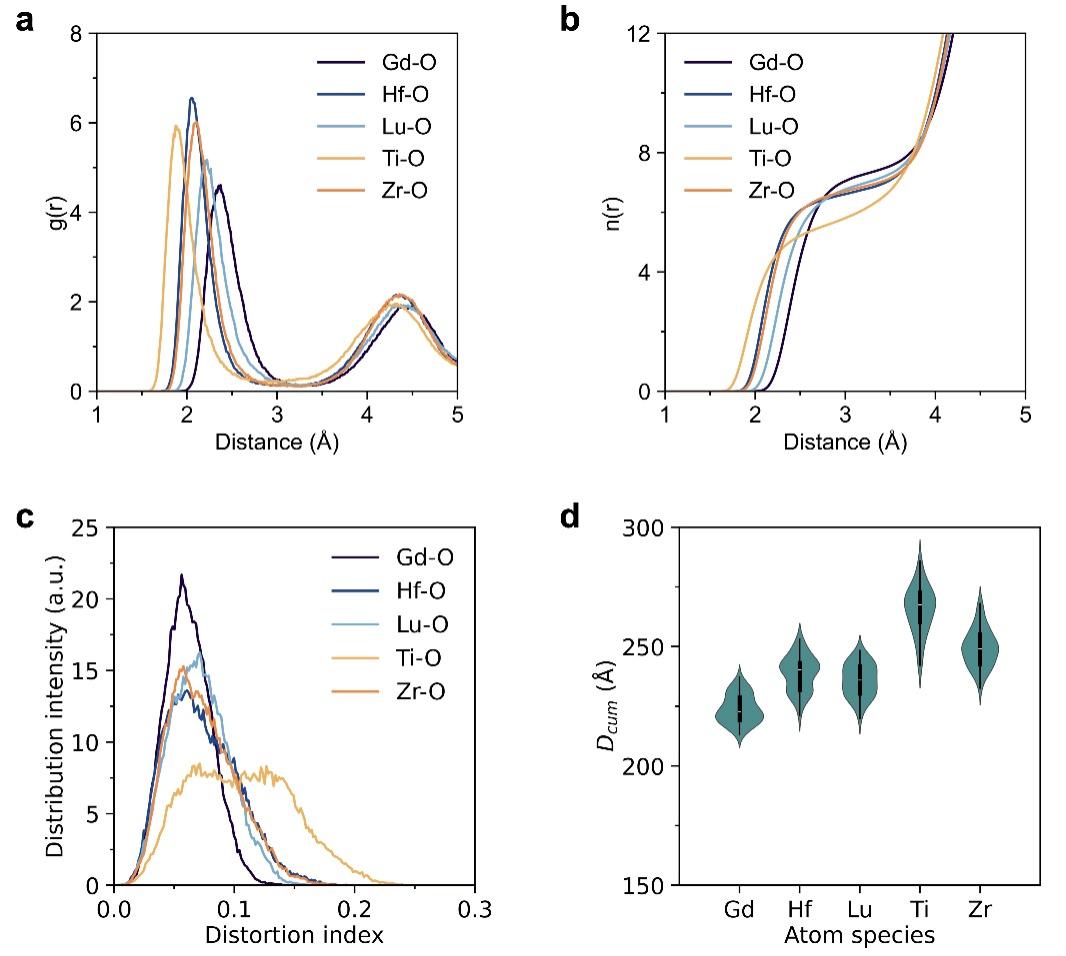


**Figure S17.** AIMD simulations of high-entropy ceramics (HEC) at 1273 K. (a) Partial pair distribution functions of the atom pairs. (b) Running coordination number of the cation. (c) Distortion index of the polyhedra. (d) Cummulative non-affine displacement of the cations.


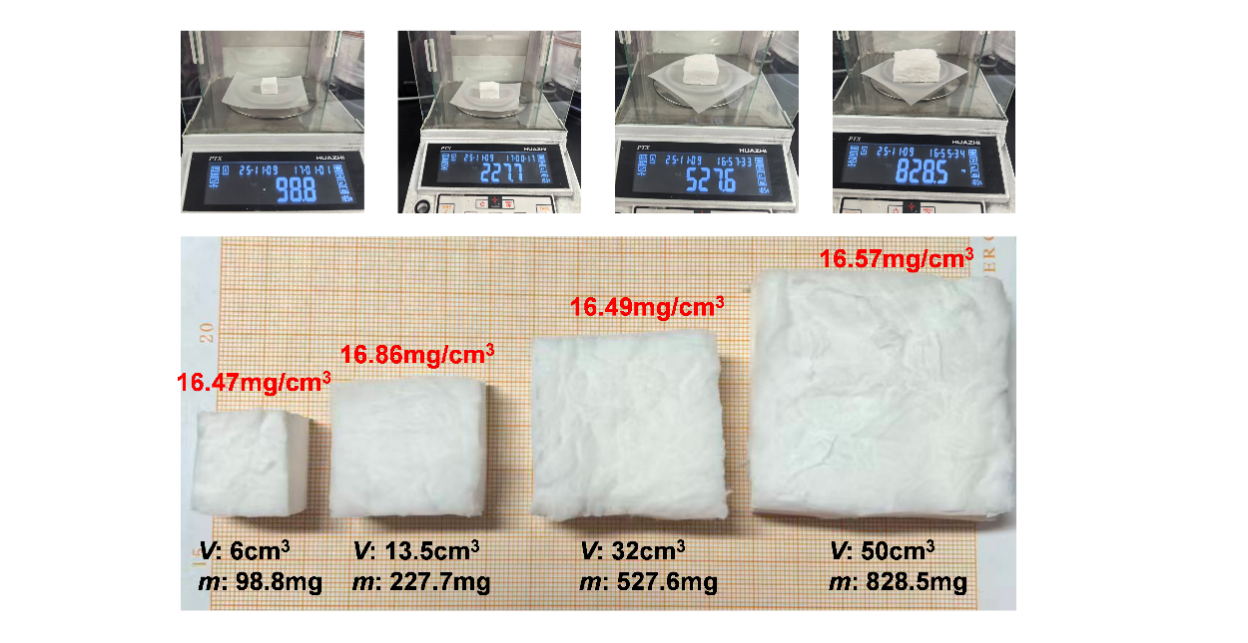


**Figure S18**. Density consistency of ZHTLG aerogels with different fabrication sizes


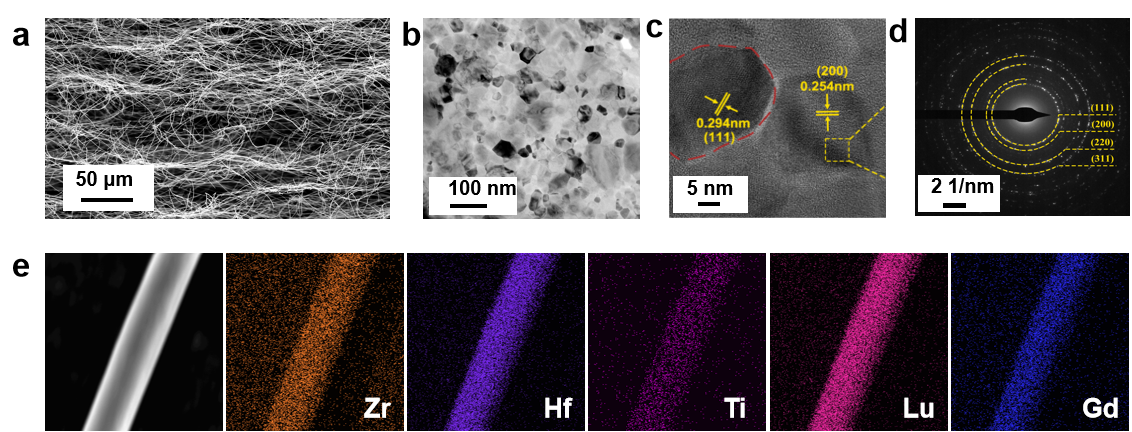


**Figure S19**. (a) The porous morphology of fiber aerogel at different magnitudes. (b)-(d) grain morphology, high-resolution TEM and selected area electron diffraction images of ZHTLG oxide. (e) The element distribution for Zr, Hf, Ti, Lu and Gd.


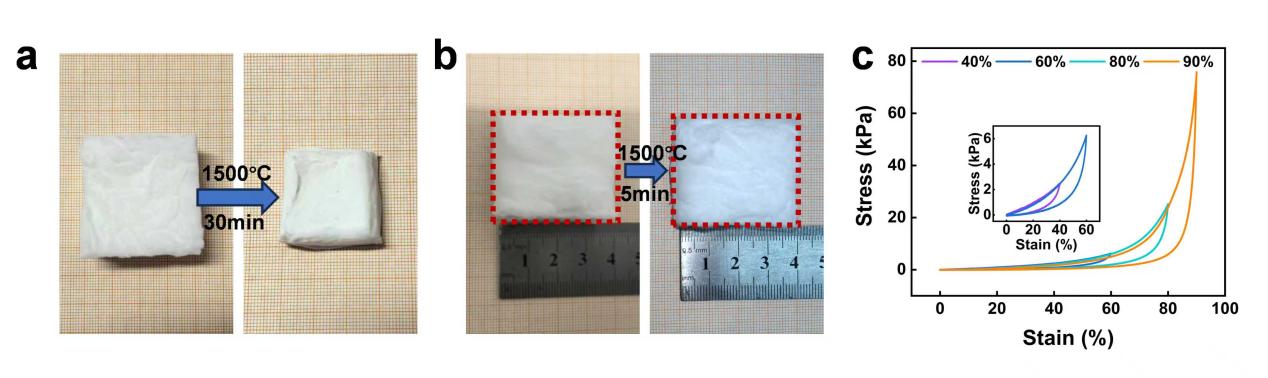


**Figure S20.** Thermal stability test of ZHTLG. (a)-(b) Morphology change of ZHTLG aerogel after heat treatments at 1500°C for 30min and 5min, respectively. (c) Stress-strain curve of ZHTLG aerogel after heat treatment at 1500°C for 5 min.

**
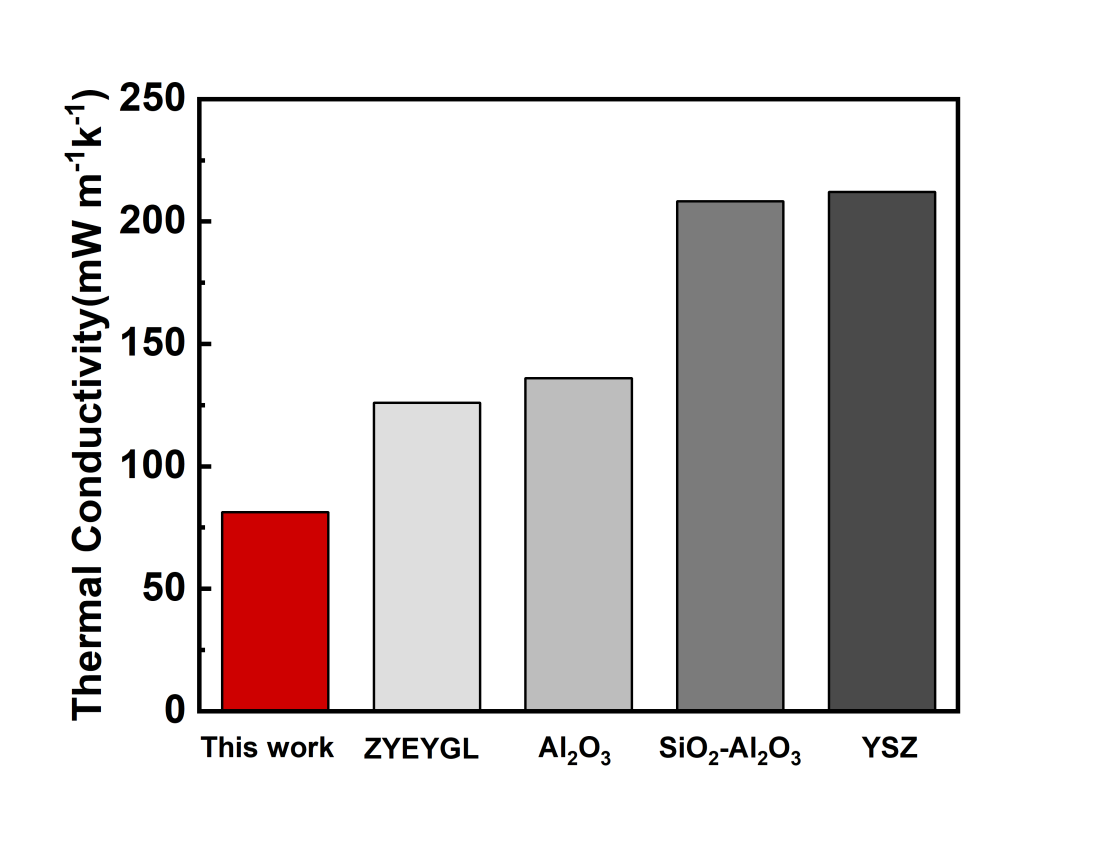
**

**Figure S21.** Thermal conductivity at 1000 °C of ZLSGY, ZYEYGL^[7]^, Al_2_O_3_^[8]^, SiO_2_-Al_2_O_3_^[9]^ and YSZ^[10]^ aerogels.

**Table S7.** Thermal conductivity at 1000 °C of ZLSGY, ZYEYGL^[7]^, Al_2_O_3_^[8]^, SiO_2_-Al_2_O_3_^[9]^ and YSZ^[10]^ aerogels.

| Materials | **This work** | ZYEYGL | Al_2_O_3_ | SiO_2_-Al_2_O_3_ | YSZ |
| --- | --- | --- | --- | --- | --- |
| Thermal conductivity (mW m^-1^K^-1^) | **81.21** | 126 | 136 | 208.25 | 212 |


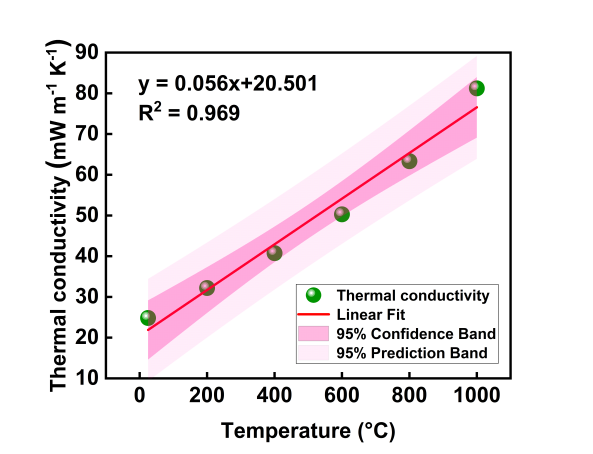


**Figure S22.** Thermal conductivity as a function of temperature with linear fitting.

**S5: Detailed description of AIMD computational models**

The partial pair distribution function *g*_𝛼𝛽_(r) between atom types 𝛼 and 𝛽 is computed by analyzing the pairwise distances over the MD trajectory. It is defined as:

$g_{\alpha\beta}\left( r \right)=\frac{\delta n_{\alpha\beta}(r)}{4\pi r^{2}\rho_{\beta}dr}$ (**3**)

where *ρ*_β​_is the number density of atom type *β*, $\delta n_{\alpha\beta}(r)$ is the number of atom type *β* between distance *r* and *r*+*dr* from an atom of type *α*. The calculation is averaged over the MD trajectory to obtain statistical results.

The running coordination number *N*_αβ_(r) is calculated by integrating the partial pair distribution function:

$N_{\alpha\beta}\left( r \right)=4\pi\rho_{\beta}\int_{0}^{r} g_{\alpha\beta}\left( r^{'} \right){r^{'}}^{2}dr^{'}$ (**4**)

A typical cutoff *r* for the first coordination shell is chosen based on the first minimum in the corresponding *g*_αβ_(r) which is 3.1 Å in this work.

The distortion index *D* is used to quantify the geometrical distortion of cation–oxygen coordination polyhedral, defined as:

$D=\frac{1}{n}\sum_{i=1}^{n} \left| \frac{d_{i}-\bar{d}}{\bar{d}} \right|$ (**5**)

where *d*_i_ is the individual bond length between the central cation and its coordinating oxygen atom, $\bar{d}$ is the average bond length, and *n* is the coordination number. A higher distortion index *D* indicates greater asymmetry in the polyhedral geometry.

The cumulative non-affine displacement *D*_cum_ is defined as the sum of the square roots of the incremental non-affine displacements $\Delta D_{i,\min}^{2}$over successive time intervals. This quantity has been widely applied to characterize the local dynamic heterogeneity and atomic mobility during the MD simulation^[11,12]^. Following the literature^[13,14]^, the non-affine displacement $\Delta D_{i,\min}^{2}$ between time *t* and *t* + $\Delta t$ is calculated as:

$D_{\min}^{2}=\frac{1}{M_{k}}\sum_{i}^{M_{k}} \left[ \boldsymbol{r}_{ik}\left( t+\Delta t \right)-\boldsymbol{J}_{k}(t)\boldsymbol{r}_{ik}\left( t \right) \right]^{2}$ (**6**)

where ***r****_ik_*(*t*) is the displacement vector between atom *i* and *j* at time *t*, ***J****_k_*(*t*) is the local strain tensor about atom *k* that minimizes $D_{\min}^{2}(k;t)$, *M_k_* is the total number of neighboring atoms around atom *k*.

$D_{cum}=\sum_{i=1}^{n} \sqrt{\Delta D_{i,\min}^{2}}$ (**7**)

where $\Delta D_{i,\min}^{2}$ is the increment of $D_{min}^{2}$ with $\Delta t$, and *n* is the total number of intervals during the AIMD equilibration.

**Reference**

[1] M. Guo, J. Du, Y. Zhang, *Aerosp. Sci. Technol.* **2024**, *145*, 108906.

[2] Y. Wang, J. He, Q. Guo, Y. Li, H. Guo, *J. Alloys Compd.* **2025**, *1010*, 178261.

[3] G. Zhou, X. Qu, L. Tong, B. Wei, Y. Sun, Z. Mou, Q. Zhang, S. Ramakrishna, Q. Meng, *Chem. Eng. J.* **2025**, *511*, 162033.

[4] X. Gan, D. Xu, Y. Lv, *Mater. Chem. Phys.* **2020**, *251*, 123111.

[5] Q. F. He, Y. F. Ye, Y. Yang, *J. Phase Equilibria Diffus.* **2017**, *38*, 416.

[6] Y. Zhao, S. Wang, B. Zhang, Y. Yuan, Q. Guo, H. Hou, *J. Solid State Chem.* **2019**, *276*, 232.

[7] S. Shang, J. Wang, M. Yuan, Q. You, Z. Song, W. Liu, X. Ye, J. Yang, S. Cui, *Mater. Charact.* **2024**, *217*, 114392.

[8] M. Gao, B. Liu, P. Zhao, X. Yi, X. Shen, Y. Xu, *J. Sol-Gel Sci. Technol.* **2019**, *91*, 514.

[9] D. Jiang, J. Qin, X. Zhou, Q. Li, D. Yi, B. Wang, *Ceram. Int.* **2022**, *48*, 16290.

[10] S. Yoon, G. D. Han, D. Y. Jang, J. W. Kim, D. H. Kim, J. H. Shim, *J. Alloys Compd.* **2019**, *806*, 1430.

[11] Z. Chen, T. Du, N. M. A. Krishnan, Y. Yue, M. M. Smedskjaer, *Nat. Commun.* **2025**, *16*, 1057.

[12] T. Du, Z. Chen, H. Liu, Q. Zhang, M. Bauchy, Y. Yue, M. M. Smedskjaer, *Mater. Today Energy* **2023**, *37*, 101390.

[13] E. D. Cubuk, R. J. S. Ivancic, S. S. Schoenholz, D. J. Strickland, A. Basu, Z. S. Davidson, J. Fontaine, J. L. Hor, Y.-R. Huang, Y. Jiang, N. C. Keim, K. D. Koshigan, J. A. Lefever, T. Liu, X.-G. Ma, D. J. Magagnosc, E. Morrow, C. P. Ortiz, J. M. Rieser, A. Shavit, T. Still, Y. Xu, Y. Zhang, K. N. Nordstrom, P. E. Arratia, R. W. Carpick, D. J. Durian, Z. Fakhraai, D. J. Jerolmack, et al., *Science* **2017**, *358*, 1033.

[14] M. L. Falk, J. S. Langer, *Phys. Rev. E* **1998**, *57*, 7192.
